# Pond water microbiome antibiotic resistance genes vary seasonally with environmental pH and tannins

**DOI:** 10.1128/spectrum.03034-24

**Published:** 2025-03-25

**Authors:** Maya Vaccaro, Andrew M. Pilat, Logan Gusmano, Minh T. N. Pham, Daniel Barich, Audrey Gibson, Mwï Epalle, Dominick J. Frost, Elianajoy Volin, Zachary C. Slimak, Chelsea C. Menke, M. Siobhan Fennessy, Joan L. Slonczewski

**Affiliations:** 1 Department of Biology, Kenyon College, Gambier, Ohio, USA; Institut Ruder Boskovic, Zagreb, Croatia

**Keywords:** antibiotic resistance, freshwater, metagenomics, environmental microbiology

## Abstract

Microbial communities of small freshwater bodies interact dynamically with environmental factors in unknown ways. Longitudinal sampling of four ponds in Knox County, Ohio, revealed relationships among antibiotic resistance genes (ARGs) and environmental factors such as pH and tannin concentrations. For each site, microbial communities were collected by filtration, and metagenomes were analyzed by short-read sequencing. ARGs were quantified using the ShortBRED pipeline to detect and quantify hits to a marker set derived from the Comprehensive Antibiotic Resistance Database. The top 30 ARGs showed increased abundance at the end of the growing season. The top two ARGs with the largest marker hits encode components of a *Stenotrophomonas* drug efflux pump powered by proton-motive force (*smeABC*) and a mycobacterial global regulator that activates a drug pump and acid stress response (*mtrA*). The *smeABC* and *mtrA* prevalence showed a modest correlation with acidifying conditions (low pH and high tannic acids). Acidity amplifies the transmembrane pH difference component of the proton-motive force, thus increasing the cell’s energy available for pump function and ARG expression. Association with microbial taxa was tested by the Kraken2/Bracken predictor of taxa profiles. The ARG profiles showed the strongest acid dependence in ponds with a high proportion of Proteobacteria, whereas a pond with high Cyanobacteria showed the lowest ARG counts. Efflux pumps such as SmeABC and transcriptional activation by MtrA incur large energy expenditures whose function may be favored at low external pH, where the cell’s proton-motive force is maximal.

**IMPORTANCE:**

Compared to rivers and lakes, pond microbial ecosystems are understudied despite close contact with agriculture and recreation. Environmental microbes offer health benefits as well as hazards for human contact. Small water bodies may act as reservoirs for drug-resistant organisms and transfer of antibiotic resistance genes (ARGs). Yet, the public is rarely aware of the potential for exposure to ARG-carrying organisms in recreational water bodies. Little is known about the capacity of freshwater microbial communities to remediate drug pollution and which biochemical factors may select against antibiotic resistance genes. This study analyzes how aquatic ARG prevalence may depend on environmental factors such as pH and tannic acid levels.

## INTRODUCTION

Ponds are defined as small, shallow bodies of water with less than 30% emergent vegetation (1–3). Studies estimate that ponds comprise 30% of the earth’s standing fresh water. Freshwater microbes play important roles as producers, consumers, and scavengers (4), and they mediate production and consumption of greenhouse gases such as methane (5, 6). Aquatic microbial communities are used as indicators of ecosystem quality (7–10). Pond systems are complex and variable; their small size means that they are more susceptible to eutrophication and changing pollutant levels from agricultural runoff. Ponds show a wide range of nutrients and chemical factors such as pH (11), tannin levels, nitrates, and phosphates (12, 13).

The variable chemistry of ponds provides the opportunity for a “natural experiment” to test which environmental variables influence the taxa profiles and prevalence of antibiotic resistance genes (ARGs). These environmental effects are concerning because aquatic microbiomes are a potential source of antibiotic-resistant organisms that increasingly threaten global public health (14, 15). ARGs in environmental microbes have the potential for transfer to pathogenic bacteria (16, 17). A modest level of antibiotic resistance is an ancient, widespread phenomenon naturally and historically occurring in all environments (18). But inputs of human origin can add substantially to the native ARG pool as well as add additional types of ARGs (19, 20). These interactions are especially concerning in agricultural and recreational landscapes. Yet, the public is rarely aware of the potential for exposure to ARG-carrying organisms in recreational water bodies such as rivers and ponds (21, 22). For example, in our region of Knox County, Ohio, previous studies report evidence of microcystins in local ponds (23) and drug-resistant *Acinetobacter baumannii* in a recreational river (24).

More needs to be learned about the capacity of aquatic microbial communities to remediate pollutants and what chemical factors may select for or against ARGs (25, 26). Especially concerning is the frontline mechanism of multidrug efflux pumps that confer resistance to multiple antibiotics (multidrug resistance, MDR). These efflux pumps are membrane protein complexes that export many substrates, including a wide array of antibiotics, metals, and metabolic byproducts from the cytoplasm (27, 28). Drug efflux spends energy, in most cases from the proton motive force (PMF), although some pump families use hydrolysis of ATP. pH plays an important role in powering MDR pumps because the transmembrane pH difference (ΔpH) is a component of PMF. At low external pH, the ΔpH is relatively large and can enhance contributions of efflux pumps (29). Pond water may be acidified by plant-derived polyphenols such as tannic acids (tannins). Alternatively, pond pH can be increased by photosynthesis of algae and Cyanobacteria. At high extracellular pH, the bacterial PMF is composed solely of electrical potential, which is partly spent maintaining an inverted ΔpH (30, 31). Thus, at high pH, we predict that bacteria will select against PMF-driven efflux pumps and large global regulons involving energy-expensive gene expression.

**Fig 1:**
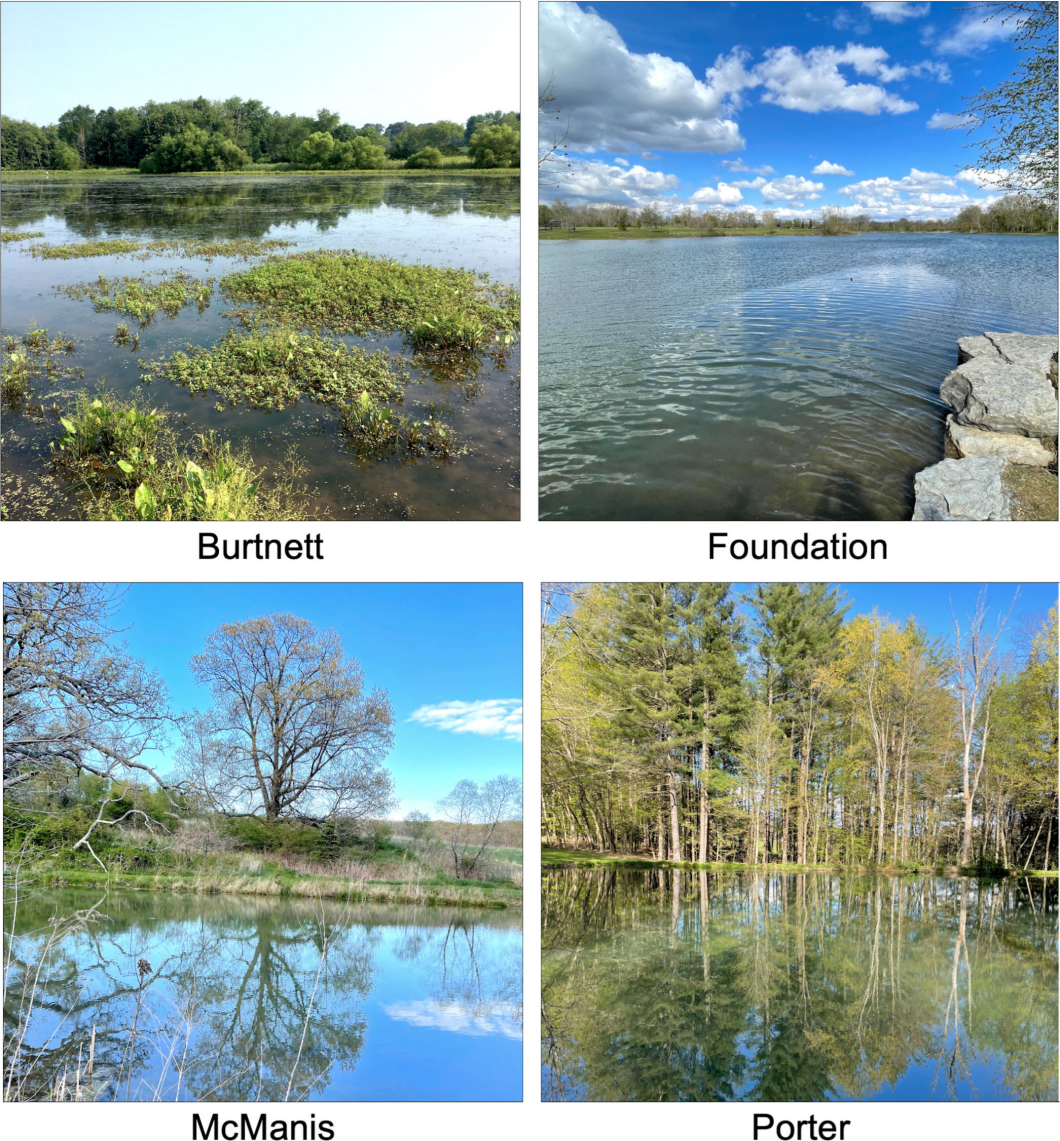
Ponds sampled during 2021 and 2022. Each small water body is located in Knox County, Ohio. See Materials and Methods for GPS locations.

Our longitudinal study was designed to reveal the changing prevalence patterns of microbial taxa and ARGs in ponds within agricultural landscapes, including the roles of pH and other chemical factors. Over two 3-month periods in 2021 and 2022, we sampled water from four ponds on or near agricultural land in Knox County, Ohio (Fig. 1). Burtnett pond is a restored pond surrounded by adjacent agricultural fields. Ariel-Foundation Park Lake 2 (Foundation pond) was formerly a silica gravel quarry. McManis pond receives fecal input from livestock. Porter pond is surrounded by pine and oak trees.

The data collected in 2021 and 2022 were treated as distinct data sets. We quantified relative ARG abundance using the ShortBRED pipeline (32) to match marker sequences from the Comprehensive Antibiotic Resistance Database (CARD) (33). We surveyed the pond microbial metagenomes for taxa markers, using Kraken2/Bracken pipelines (34–36). We characterized the relationships of ARG prevalence with pond chemistry and taxa, with a particular focus on the role of pH and tannin concentration.

## RESULTS

### Ponds' physical traits and chemistry

**Fig 2:**
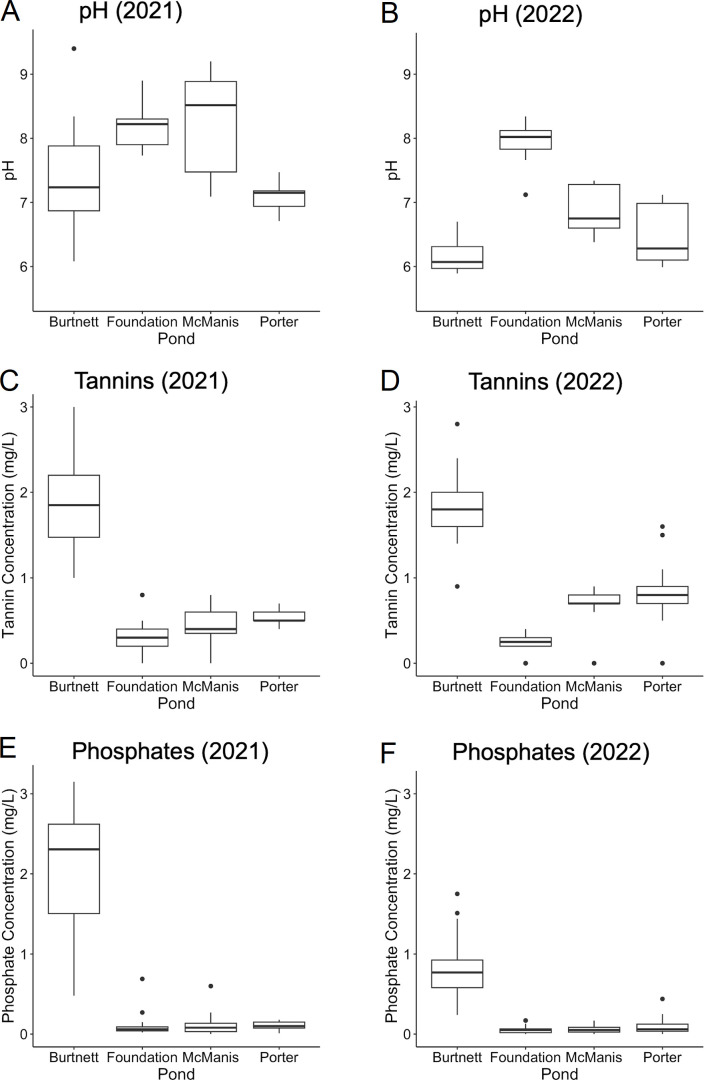
Average levels of pH, tannins, and phosphate for each pond. Values of pH, tannin concentration (mg/L), and phosphate concentration (mg/L) were compiled across each sampling year: 2021 (A, C, and E) and 2022 (B, D, and F).

Water samples were obtained from the four ponds during two seasons, in 2021 and 2022, as described in Materials and Methods. All metadata are presented in Tables S1 and S2. The ponds varied in their water chemistry, including pH, tannin concentration, and phosphate levels (Fig. 2). The pH levels varied widely among the ponds (Fig. 2A and B). The lowest pH was found in Burtnett pond, especially in 2022. Burtnett had much greater levels of tannins than other ponds (Fig. 2C and D) as well as higher dissolved phosphate (Fig. 2E and F) and dissolved organic carbon (DOC) (Table S5). In Foundation pond, the conductivity (150–670 μS/cm) was consistently higher than in the other three ponds (100–300 μS/cm), suggesting slightly higher salinity. McManis and Porter ponds had variable levels of pH, with low levels of tannins, phosphate, and ammonia.

**Fig 3:**
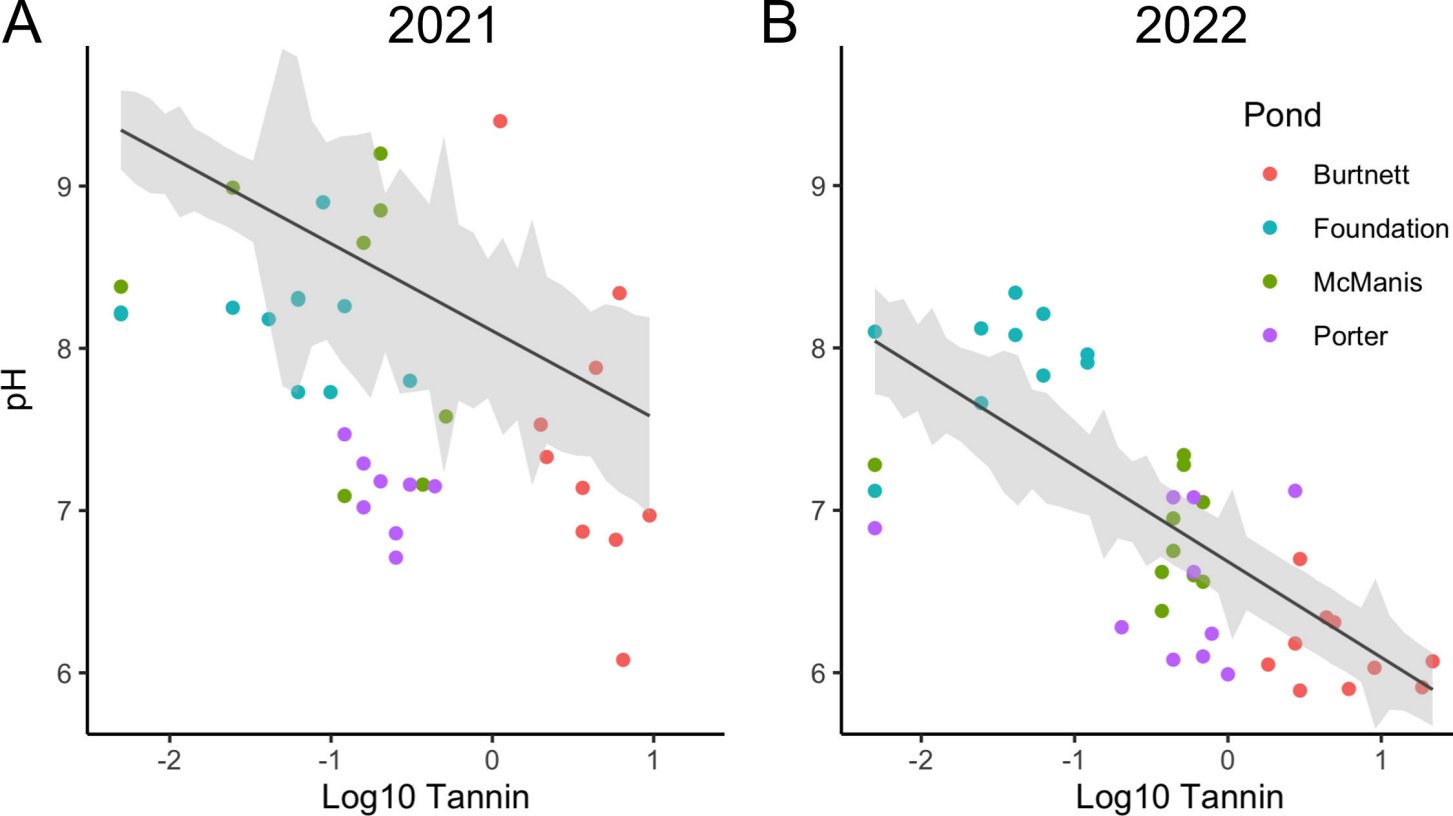
Plots of pH against log_10_ tannin concentration. Tannin concentration and pH are moderately correlated for both sampling periods (2021: r^2^ = 0.1944, *P* < 0.01; 2022: r^2^ = 0.4139, *P* < 0.001). Symbols indicate the following: orange (Burtnett pond), cyan (Foundation pond), green (McManis pond), and purple (Porter pond). Data are from 2021 (A) and 2022 (B).

Tannic acids dissociate with a pK_a_ of approximately 6 and thus can acidify aquatic systems (37). We found strong negative correlations between pH and tannin levels (Spearman correlation *R* = −0.46, *P* < 0.001, for 2021; *R* = −0.73, *P* < 0.001, for 2022). The pH and tannin levels are plotted in Fig. 3. The association of low pH with high tannin levels appears especially strong for Burtnett pond, where tannin levels were consistently high.

**Fig 4:**
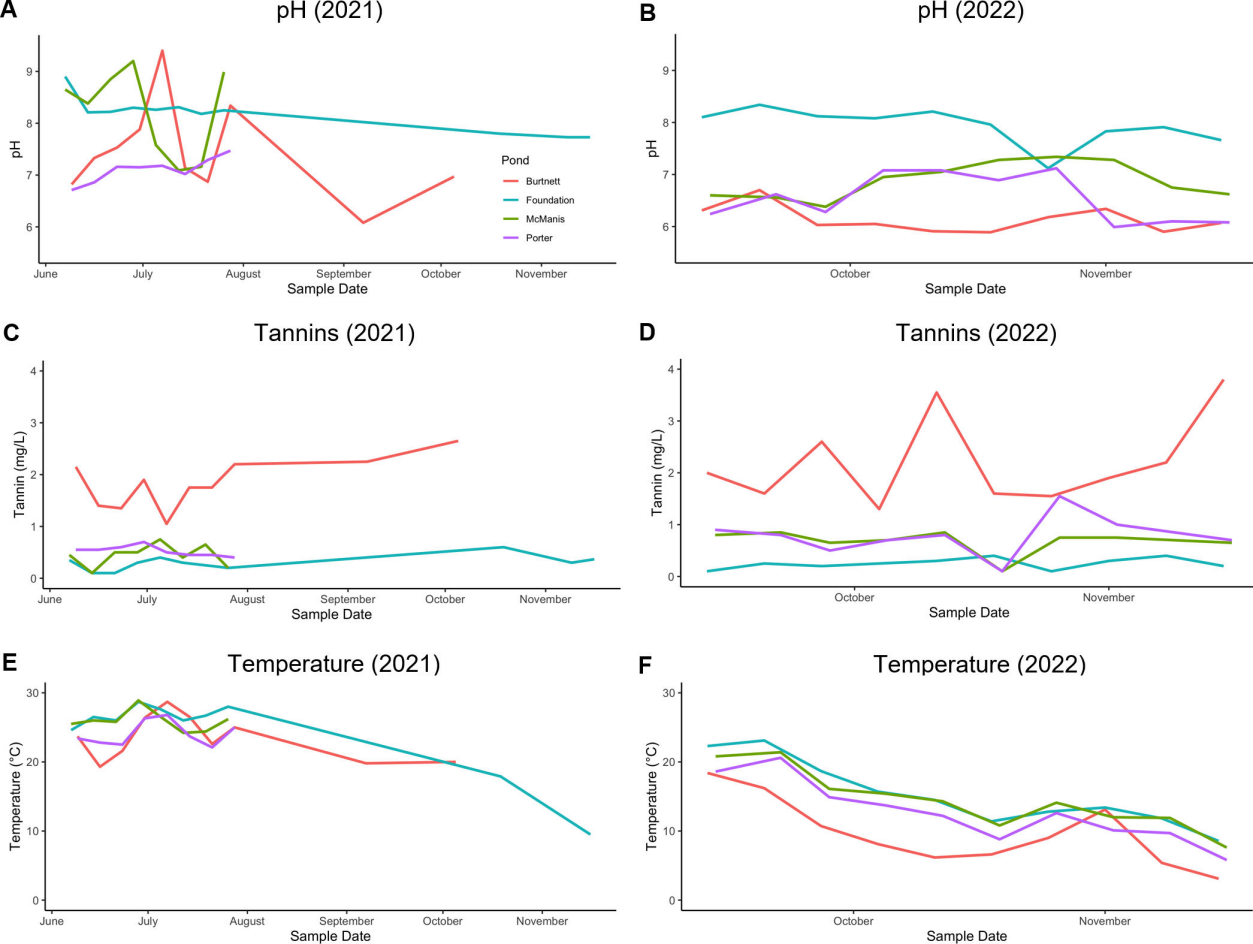
Time course of pond water measurement of pH (A, B), tannin levels (C, D), and water temperature (E, F). Line colors represent the following: orange, Burtnett; cyan, Foundation; green, McManis; and violet, Porter. Data are from 2021 (A, C, E) and from 2022 (B, D, F).

The acidity and tannin levels varied over the seasons (Fig. 4). In 2021, Burtnett pond pH levels fluctuated over a range of pH 6–9. From September through November of both years, pH was below 7 (pH 5.9–6.3). By contrast, Foundation pond maintained higher pH than Burtnett, always above 7 (pH 7.1–8.9) and higher than all other ponds in the fall months of 2022. Tannins reached the highest levels in Burtnett pond. Other ponds showed some increase in tannins in the fall, though never as high as Burtnett. The ponds also showed seasonal variation in temperature, with water temperatures declining from summer to fall (Fig. 4E and F).

### The ponds had distinctive patterns of ARG relative abundance

Environmental bacteria possess a wide range of defenses against antibiotics, including broad-substrate efflux transporters as well as highly specific drug pumps and enzymes that modify the drug or its substrate (14, 18). The genes encoding many of these are compiled in the CARD database (33). We detected ARGs from CARD in our metagenomes using a marker set constructed by the ShortBRED pipeline (32) (Table S3). ShortBRED converts CARD sequences into homologous protein families and searches for unique motifs to use as unique and selective “markers” for each protein family. The unique protein motifs are then screened against all the proteins in the universal UNIPROT database that are not listed in CARD. Nonspecific markers are thus eliminated. All markers in Table S3 were derived from the CARD database, accessed on 06/14/2021 (see Materials and Methods). Because of the UniProt sequence filtration, we cannot assume that all ARGs were identified from the metagenome; however, those identified are highly specific.

**Fig 5:**
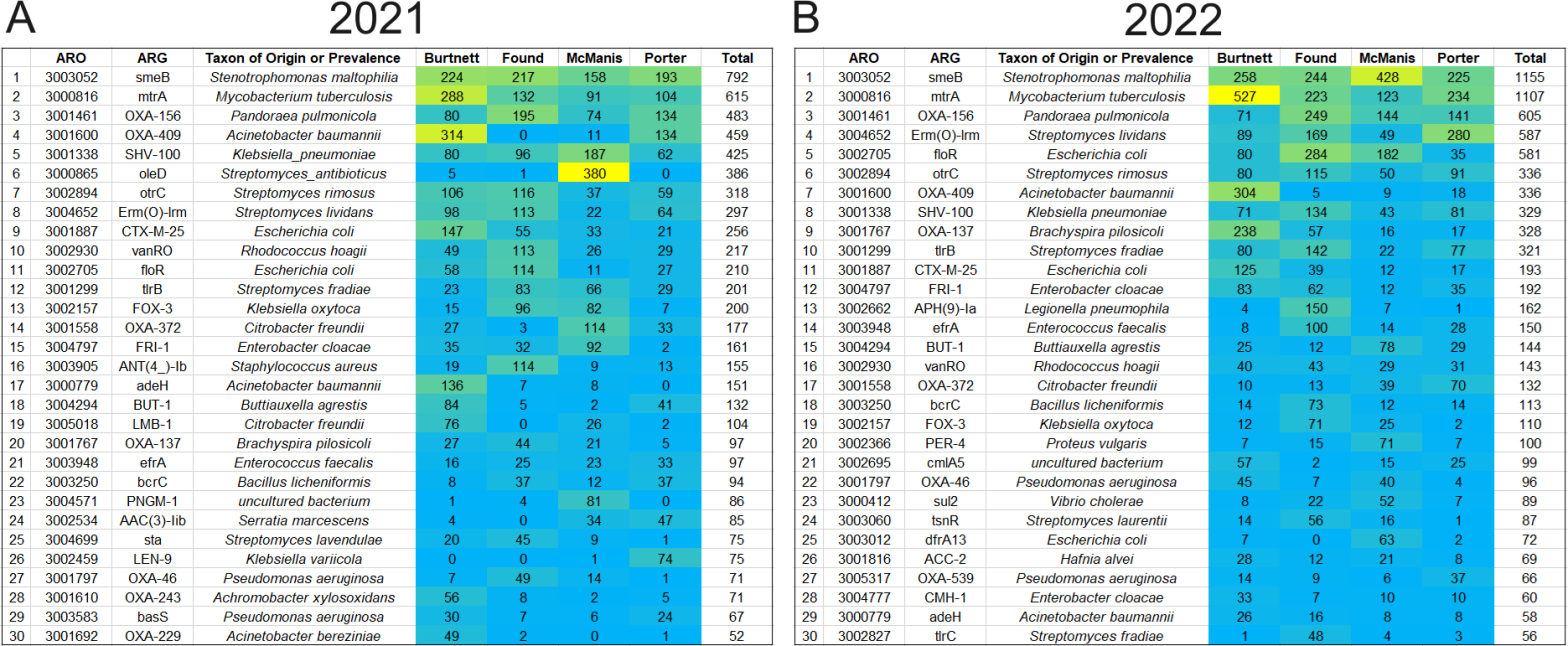
Antibiotic resistance genes (ARG hits) predicted by ShortBRED markers. Relative abundance of top 30 ARG marker hits is shown. Read hit numbers are ranked in descending order by total hits across all samples. The heat map shows relative abundance of ARG hits in each pond sampling location. Yellow, high values and cyan, low values. Data are from 2021 (A) and 2022 (B).

The read hits for each ARG were compiled from all samples for each year, 2021 and 2022. Using ShortBRED, we counted ARG marker hits from all sample dates; for nearly all dates, two independent samples were analyzed. In both years, the three most prevalent ARGs were *smeB*, *mtrA*, and OXA-156 (Fig. 5). The *smeB* gene encodes a subunit of the SmeABC multidrug efflux pump of *Stenotrophomonas maltophilia*, an opportunistic pathogen (38, 39). The *mtrA* gene is a global response regulator found in environmental species of *Mycobacterium* and *Corynebacterium* as well as *Mycobacterium tuberculosis* (40, 41). MtrA not only confers intrinsic drug resistance but also mediates response to acid stress (42). The third-ranked ARG in our ponds, OXA-156, encodes a beta-lactamase, one of many found in environmental microbiomes (43, 44).

Other ARGs showed variable ranking depending on the pond. The beta-lactamase OXA-409 was highly prevalent in Burtnett pond and absent or barely present in other ponds. The macrolide glycotransferase gene *oleD* showed high prevalence in McManis pond in 2021 but did not show up in 2022. Such findings would be expected for the patchy nature of pond water systems with high amounts of suspended particulates.

### Relative abundance of certain ARGs showed correlations with acidity and tannin concentration

**Fig 6:**
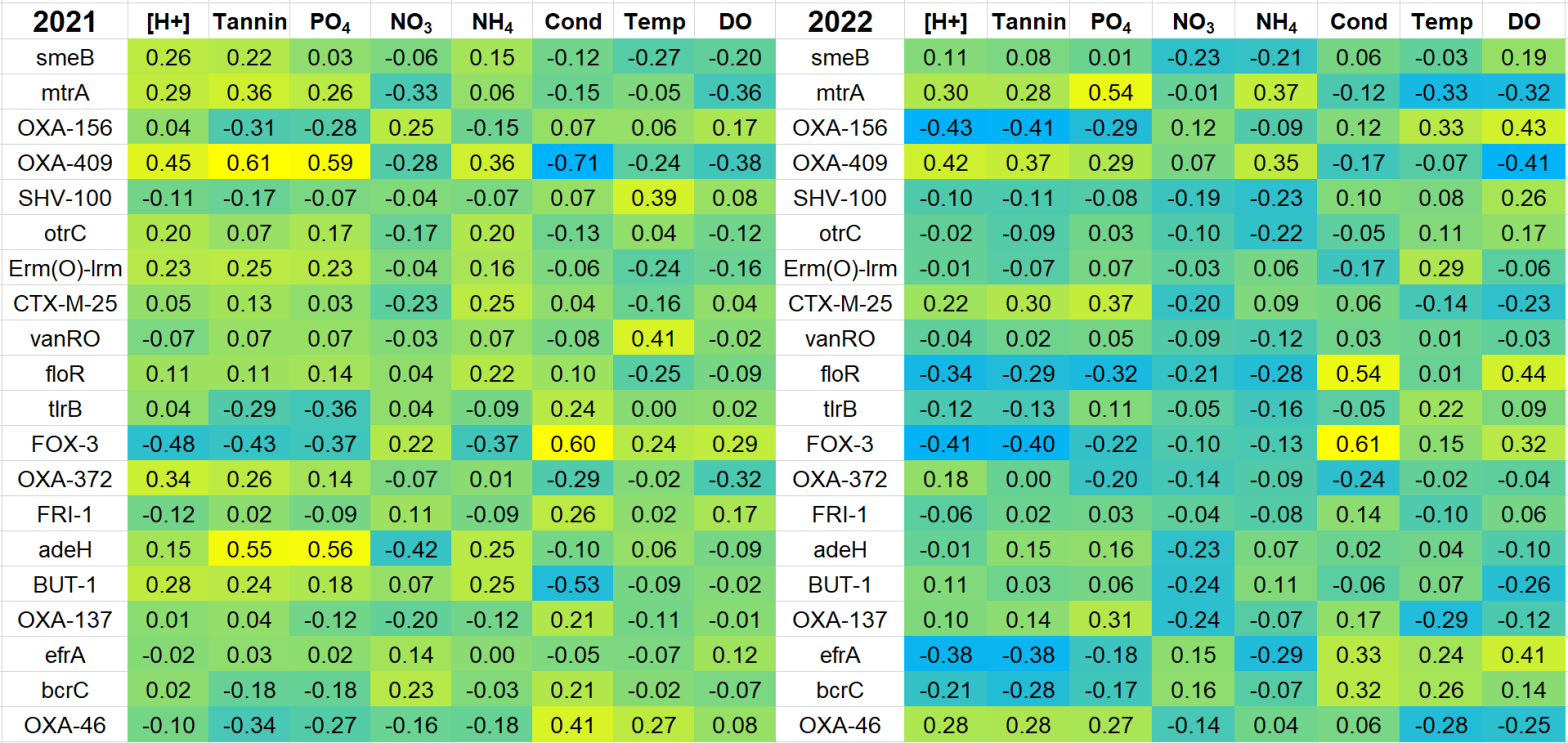
Heat map of Spearman correlations between top-ranked ARGs and chemical measures. The 20 ARGs shown are those that appeared in both 2021 and 2022 among the top 30 ranked markers for each year. Cell color indicates the range of Spearman *R* values from positive (yellow) to negative (cyan). Chemical and physical measures include acidity, tannin concentration (mg/L), phosphate concentration (mg/L), nitrate concentration (mg/L), ammonia concentration (mg/L), conductivity (μS/cm), water temperature (°C), and dissolved oxygen (DO) (mg/L). *P* values are provided in Table S4.

We tested the hypothesis that relative ARG abundance in the pond microbial communities is associated with various chemical factors such as pH and tannin concentration. For a meaningful comparison, we compiled a list of ARGs that were found in the top 30 ranked ARGs in both 2021 and 2022 (Fig. 5). This list of top-ranked ARGs shared by both years (20 in all) was tested using Spearman correlations between marker hit numbers and measures of pond chemistry (Fig. 6; *P* values in Table S4). Ten of the 20 top-ranked ARGs (*smeB*, *mtrA*, OXA-409, *otrC*, *Erm(O)-lrm*, *CX-M-25*, *floR*, OXA-372, *adeH*, and BUT-1) showed a positive correlation with acidity and tannin concentration in 2021. Of these ARGs, *smeB*, *mtrA*, OXA-409, CTX-M-25, and OXA-372 also showed positive correlation with acidity and tannins in 2022. The differences between the two years may be associated with the high variance of many pond water factors and with the seasonal difference of the sample ranges (June through November 2021, September through November 2022). For example, the summer season of samples in 2021 showed generally higher pH values (Fig. 4).

**Fig 7:**
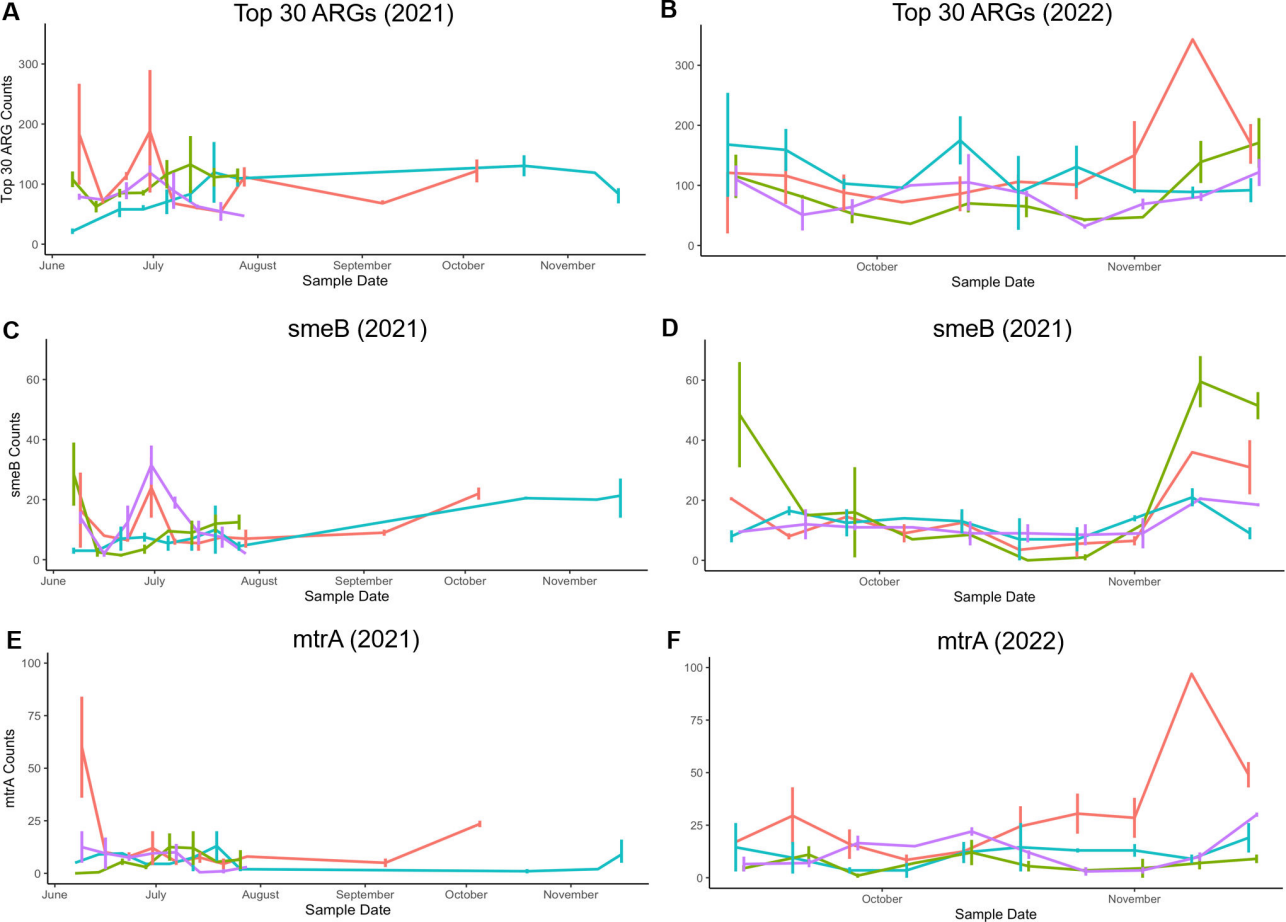
Time course of ARG hits for the sum of top 30 ranked ARGs (A, B), *smeB* (C, D), and *mtrA* (E, F). Line colors represent the following: orange, Burtnett; cyan, Foundation; green, McManis; and violet, Porter. Data are from 2021 (A, C, E) and from 2022 (B, D, F).

To investigate the roles of pH and tannins in ARG abundance, we separated the ARG counts among the four ponds. We then plotted pond ARG relative abundance over the course of time (Fig. 7), in parallel with the time course of pH levels, tannin concentration, and temperature (Fig. 4). During the fall of 2022, three of the four ponds had pH values generally below pH 7, and in Burtnett pond, the tannins reached a high concentration. In Burtnett, the same period in the fall saw an increase in levels of *smeB*, *mtrA*, and the top 30 ranked ARGs. The Burtnett *smeB* and *mtrA* counts showed weak correlations with pH (*smeB*: *R* = −0.18, *P* = 0.27; *mtrA*: *R* = −0.39, *P* = 0.02) and strong correlations with tannin concentration (*smeB*: *R* = 0.55, *P* < 0.001; *mtrA*: *R* = 0.33, *P* = 0.04). These correlations suggest a connection between acidity (conferring high proton motive force) and drug resistance mechanisms that are energy-expensive.

Tannins are a component of overall DOC which might affect ARGs by providing substrate that increases respiration (45, 46). During the 2022 sampling, DOC was measured for alternate samples. DOC levels were generally higher in Burtnett pond than in the other three ponds (Table S5). DOC levels showed a significant correlation with pH overall (*R* = −0.64, *P* < 0.001) but not with pH in Burtnett pond, the source of most pH variation (*R* = −0.13, *P* = 0.74). Only OXA-409 and OXA-46 showed positive correlations with DOC.

Another factor that changed over time was water temperature, which declined overall from summer through fall (Fig. 4E and F). However, only modest correlations were found for water temperature and ARG relative abundance (Fig. 6).

Phosphate was also investigated as a factor in ARG relative abundance. Two of the highly ranked ARGs, *mtrA* and OXA-409, showed positive correlation with phosphate levels in both 2021 and 2022 (Fig. 6). These two ARGs were found predominantly in Burtnett pond, where phosphate was high (Fig. 2). Nevertheless, within the Burtnett data set combining 2021 and 2022, the two ARGs showed no significant positive correlation with phosphate level (*mtrA*: *R* = −0.44, *P* = 0.005; OXA-409: *R* = 0.25, *P* = 0.12).

### The ponds had distinct patterns of microbial taxa, which varied over time

**Fig 8:**
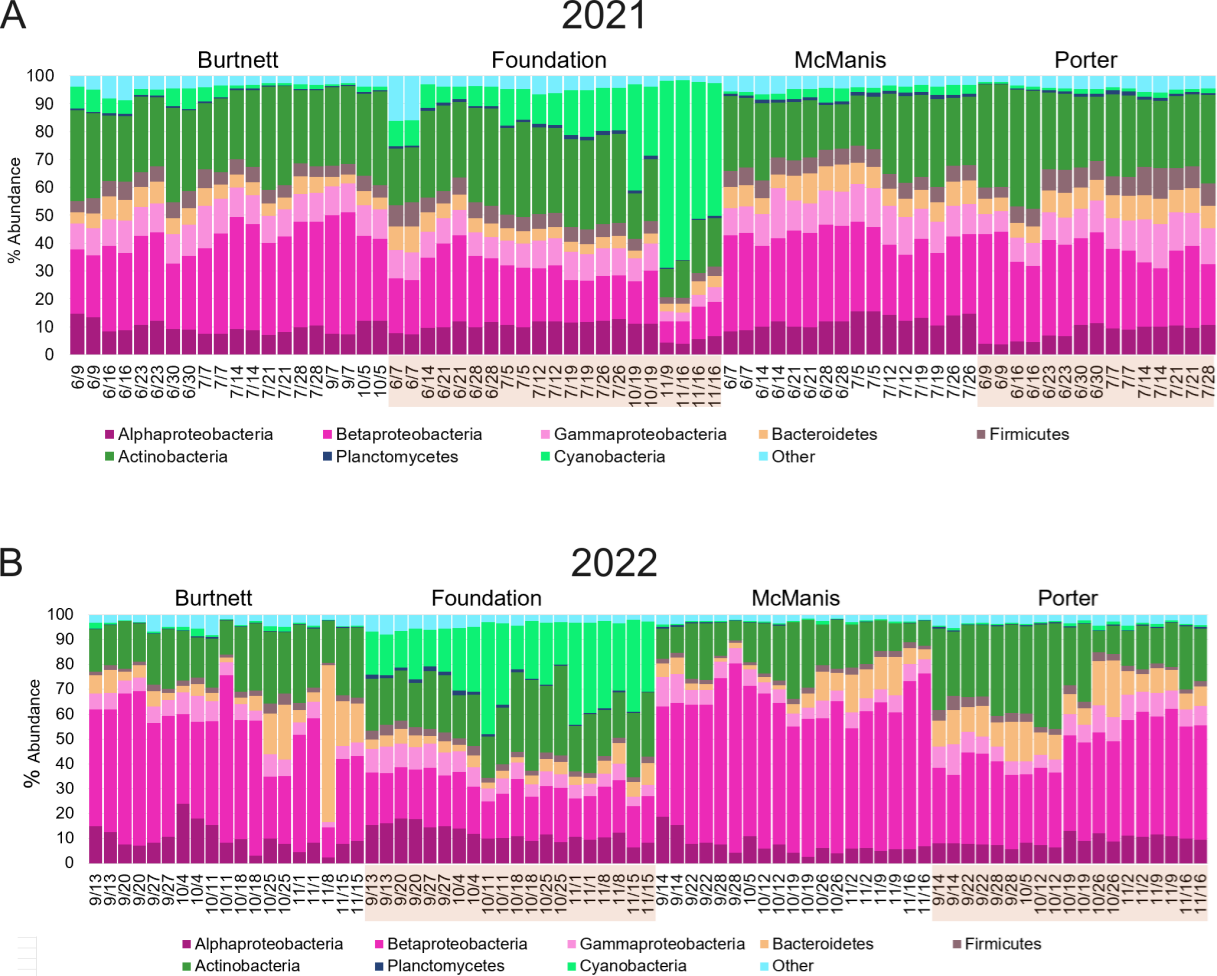
Taxa relative abundance: major bacterial phyla and classes predicted by Kraken2/Bracken in each pond across sampling dates. Taxa that make up less than 1% of the overall sample are grouped together and labeled as “Other.” (A) Samples from 2021 and (B) samples from 2022.

Overall, 60% of the top 30 ARGs are found in species of Gammaproteobacteria (Fig. 8). We examined the taxa profiles of our ponds. To predict the relative abundance of microbial taxa in each sample, we applied the Kraken2/Bracken pipeline (34) to our 150 bp read metagenomes (Fig. 8). Kraken2 assigns taxa to short sequences by *k*-mer alignment to a reference database, while Bracken then uses taxa assignments to estimate the relative abundance of taxa in the sample. Using these pipelines, we obtained consistent estimates of bacterial taxa, though not for archaea or eukaryotes, whose marker representation was limited in the database (see Materials and Methods).

In both the 2021 and 2022 metagenomes, the taxa Actinobacteria and Betaproteobacteria showed high relative abundance across all ponds and seasons (Fig. 8). At the genus level (Fig. S1), the major predicted Actinobacteria were *Planktophila* and *Nanopelagicus*. These genera are chemoheterotrophs that are commonly found in freshwater communities (47, 48). The major Betaproteobacteria predicted were *Polynucleobacter*, *Limnohabitans* (49, 50), and *Methylopumilus* (51). These organisms are typical of high-quality freshwater systems. *Polynucleobacter* species commonly feed on algal products (50). *Methylopumilus* and related methylotrophs oxidize single-carbon compounds such as methylamine, an important niche in water and soil environmental ecosystems (51).

The Foundation pond showed taxa patterns that were distinct from those of the other three ponds. Cyanobacteria were highly abundant in the fall months (September through November) (Fig. 8). The 2021 data set showed a steady increase in the proportion of Cyanobacteria across the summer and fall months. The genera of Cyanobacteria shifted from the picocyanobacteria *Synechococcus* in the summer to the filamentous *Planktothrix* in the fall (Fig. S1). *Synechococcus* species are important oxygenic phototrophs (52) although blooms may produce toxins (53). Seasonal succession of aquatic Cyanobacteria is an important factor in environmental quality (54, 55); *Planktothrix* can contribute to toxin-producing blooms (56, 57). Besides phototrophs, Foundation samples from the summer months of 2021 showed distinctive genera of Actinobacteria (*Nanopelagicus*) and Betaproteobacteria (*Methylopumilus*). Thus, Foundation pond showed succession of prevalent taxa from *Nanopelagicus*, *Methylopumilus*, and *Synechococcus* in the summer to *Planktothrix* in the fall.

### pH and tannins drive microbial community structures

To determine how pond chemistry associates with taxa prevalence, we performed Nonmetric Multidimensional Data Scaling (NMDS) (58). The NMDS algorithm iteratively searches for the best placement of data in a *k*-dimensional space given only the ranked similarity of communities (59, 60).

**Fig 9:**
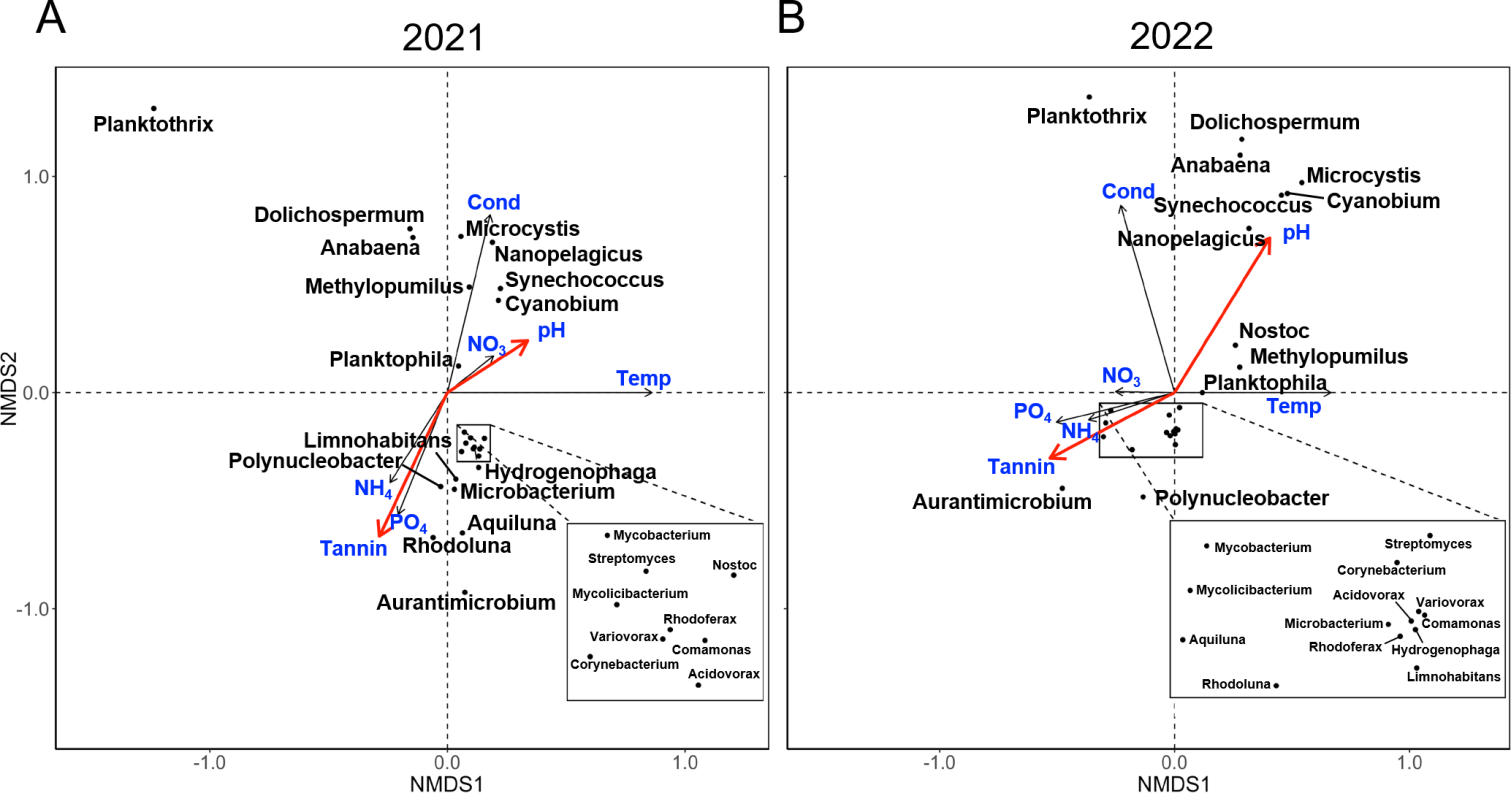
Correlation of microbial taxa with environmental factors. Ordination plot was obtained by Nonmetric Multidimensional Data Scaling (NMDS) (59–61). Arrows represent strength of abiotic factors associated with community structure. Arrows in the inverse direction represent a negative association. (A) 2021 sampling period; (B) 2022 sampling period. Cond, conductivity; Tannin, tannin concentration; and Temp, water temperature (°C).

Certain taxa were clustered according to the pH/tannin axis, both in 2021 and 2022 (Fig. 9). Cyanobacteria such as *Cyanobium*, *Synechococcus*, and *Microcystis* were generally associated with a slightly alkaline pH and lower tannin concentration. These conditions were most represented by the Foundation pond in the fall, conditions where ARG counts were low.

Many Actinobacteria (such as *Streptomyces*, *Mycobacterium*, and *Aurantimicrobium*) as well as Betaproteobacteria (*Polynucleobacter* and *Limnohabitans*) were more prevalent in acidic, high tannin conditions. Tannins were highly associated with the relative abundance of *Aurantimicrobium* and *Polynucleobacter*. While high tannin loads have been previously found to correspond with *Polynucleobacter* species (62), our study additionally associates tannins with *Aurantimicrobium* (Spearman *R* = 0.43, *P* = 0.43).

Cyanobacteria (*Planktothrix*, *Anabaena*, *Synechococcus*, and *Cyanobium*) also clustered with higher conductivity levels. When the two data sets were aggregated, the relative abundance of *Planktothrix* showed a clear negative correlation with pH; that is, increased levels with acidity (Spearman *R* = −0.76, *P* < 0.01). No effect of phosphate level was seen (*R* = −0.05, *P* = 0.74), which has previously been associated with *Planktothrix* blooms (63).

## DISCUSSION

The factors driving seasonal ARG prevalence in ponds and lakes are poorly understood. A review of ARG prevalence in Chinese lakes finds high ARG abundance under eutrophic conditions, but does not find consistent correlations with factors such as pH or temperature (26). A seasonal study of an agricultural pond finds no correlation of ARGs with physical factors but an uptick of ARG diversity following a precipitation event (64). A study of an agriculturally disturbed lake finds higher sulfonamide resistance genes in spring than in fall, but lower tetracycline resistance (65). A study of urban environmental water finds peak abundance and diversity of ARGs in the summer (66). With all the studies published on aquatic systems, there are no general models of ARG prevalence and environmental factors.

For our investigation of microbial community composition and ARG abundance, the four ponds offered a natural experiment in their shared location with similar geology and agricultural proximity, while showing some variety in chemical and physical factors (Fig. 1, 2, and 4). Burtnett pond had significantly higher levels of tannic acids and phosphate than the other three ponds, whereas Foundation pond had higher pH values. In addition, Foundation pond had higher conductivity values (Tables S1 and S2), which may indicate a higher salt content.

The ponds showed interesting variation over the seasons. Certain ARGs (*smeB* and *mtrA*), as well as the top 30 ARGs in 2022, reached their highest counts during late November, at approximately the time of greatest acidification (Fig. 7). *S. maltophilia* with *smeB* has been found as a contaminant in drinking water (67) and is a member of a genus widespread in environmental sources such as soil and plants (68). The MtrAB two-component regulatory system governs cell morphology; its loss increases sensitivity to vancomycin and rifampicin but decreases sensitivity to isoniazid. Its role in mediating acid resistance would be consistent with its increased finding in environments at low pH (42).

For some ponds, the seasonal variation in ARGs could be connected with pond acidity and tannins. In Burtnett, the level of tannins rose in the fall months during tree leaf senescence and leaf inputs to the pond (September through November). Porter pond, in 2022, had a slight rise in tannins as well as a decline in pH. In general, the late fall was associated with acidification, which could be associated with influx of tannic acids from fallen leaves.

The highest ARG counts were found in the fall of 2022 in the three ponds where pH was low and (in Burtnett) tannins were high. All ponds had high proportions of Actinobacteria and Betaproteobacteria (Fig. 5), which commonly occur in high-quality environments with minimal human disruption (47, 50). Within these broad taxa categories, the ponds showed interesting differences in genera; Foundation had a higher proportion of *Nanopelagicus* species, whereas McManis had a higher amount of *Planktophila* (Fig. S1). The presence of the Betaproteobacterium, *Methylopumilus*, shows a capacity for turnover of reduced one-carbon compounds such as methanol and methylamine, an important role in aquatic carbon cycles (69). In Foundation, the succession of Cyanobacteria from *Synechococcus* in the summer to *Planktothrix* in the fall is important for relevance to oxygen production but also the potential for microcystin-producing blooms (54, 56).

Our pond microbiomes showed various ARGs that are found in environmental taxa such as *Stenotrophomonas*, *Mycobacterium*, *Pandorea*, *Streptomyces*, and *Acinetobacter*. While harmless for most people, many environmental microbes are now showing up as opportunistic pathogens of immune-compromised patients. This is concerning, for example, for *S. maltophilia* (39) and for non-tuberculosis *Mycobacterium* species (70), which are sources of the top two ARGs found in our samples (*smeB* and *mtrA*, respectively).

The prevalence of ARGs in freshwater bodies is subject to numerous factors, most importantly the influx of human and agricultural sources of drug-resistant bacteria (16, 20). At the same time, the environmental communities that receive such inputs possess some resilience and ability to outcompete drug-resistant organisms. For example, our study of river ARGs associated with wastewater showed that the ARG counts largely decreased in river water several kilometers downstream of the plant (24). Thus, it is of interest to consider which environmental factors might affect the resilience of water bodies receiving ARG inputs.

In our present study, we show modest evidence that the abundance of certain ARGs depends upon water pH, with possible enhancement by acidification associated with tannin inputs. It would be informative to follow up this evidence with controlled experiments in microcosms to assess the magnitude of the pH effect and determine whether it is mediated by energetics. Energy cost is an important trade-off of ARGs, so it would be good to know how this factor influences the environmental prevalence of drug-resistant bacteria.

## MATERIALS AND METHODS

### Water sampling and metadata

Water was sampled from four ponds around Knox County, Ohio, a historically agricultural county with a scenic river and one small city (Mount Vernon, pop. 17,000) (Fig. 1). While none of the four sampling sites was used directly in agriculture, each was located near agricultural land. Burtnett pond (40°20′58″ N, 82°19′31″ W) was a restored wetland with a high abundance of duckweed and cattails and high traffic by geese and other waterfowl. It was surrounded by soybean fields and sheep pastures. The water had high concentrations of tannin and phosphate. Foundation pond (40°23′10″ N, 82°29′49″ W) was a restored quarry for silica gravel formerly used by a glass factory. The pH was consistently high (pH 7.1–9.0), and the water was clear with a large population of geese and minimal input from surrounding vegetation. Porter pond (40°22′21.1188″ N, 82°24′56.97″ W) had heavy input of leaf litter from oak, maple, and pine trees. McManis pond (40°23′53″ N, 82°24′24″ W) had vegetation including cattails, weeping willows, and duckweed with fecal input from poultry and goats.

Water microbiomes were obtained and analyzed using methods based on reference 24. Pond water was sampled weekly from June to July 2021 and between September and November 2021. A second data set was collected from each pond weekly from September to November 2022. All analyses were performed separately on each of the two data sets, using the same methods for both.

The values of pH, conductivity (μS/cm), temperature (°C), and dissolved oxygen (DO) concentration (mg/L) were measured in the field using a Hannah pH/conductivity combination meter and a YSI Pro20 DO meter (Yellow Springs Instruments, Yellow Springs, OH, USA). Four 1 L samples were collected from each site between 9:00 and 11:00 a.m. and kept on ice in Whirl-Pak bags. Once in the lab, 100 mL of each sample was filtered using a sterile 0.22 µm Microfil V filter to collect microbial samples. During our sampling period in 2021, we switched to acid-washing the outer plastic filter from the Microfil filters and using sterile 0.22 µm MF-Millipore filters in the plastic casings. Filter samples were stored in sterile 2-mL microfuge tubes at −80°C until processing. All colorimetric analyses were done within 48 h of sample collection using a Hach DR900 multiparameter portable colorimeter. Test ‘N Tube kits were used to measure orthophosphate, low range ammonia, low range nitrate, and tannin concentration in mg/L using the protocol outlined in the kits (Hach, Loveland, CO, USA). Low range nitrate was analyzed using the cadmium reduction method, and tannin concentration was evaluated using the tyrosine method. Reusable glassware for colorimetry was placed in 10% HCl acid wash overnight and rinsed with deionized water between uses. For the 2022 data set, DOC was measured by combustion analysis (UC Davis Analytical Lab). Water samples (50 mL) were acidified to remove inorganic carbon, then injected into the high-temperature combustion reactor with an oxidative catalyst. Following oxidation to completion, the CO_2_ was measured at 4.2 µm by infrared detection.

### DNA isolation and sequencing

From each pond and sample date, two filter samples were processed for DNA sequencing. Metagenomic DNA was isolated using the ZymoBIOMICS DNA mini-prep kit, as described (24). For each set of preps, 75 µL of the ZymoBIOMICS microbial community standard was prepared under the same conditions to serve as a control. This mock community contained defined proportions of 10 microbes (5 Gram-positive bacteria, 3 Gram-negative bacteria, and 2 fungal microbes).

Purity of DNA samples was determined using Nanodrop analysis (Thermo Fisher Scientific). Admera Health performed library construction with Nextera XT library kit (Illumina, San Diego, CA, USA) using Illumina 8-nt dual indices, following the manufacturer’s recommendations. Amplifiable molar concentration of each library was measured by KAPA SYBR FAST qPCR with QuantStudio 5 System (Applied Biosystems, California, USA). Sequencing was performed on an Illumina NovaSeq S4 (Illumina, California, USA) with a read length configuration of 150 PE for 40 M PE reads (20 M in each direction) for each sample (Admera Health, New Jersey, USA).

### ARG marker analysis

Metagenomic short reads were matched to ARG markers generated by ShortBRED-Identify (32) using the updated CARD 3.1.2 database of ARGs (33) (https://card.mcmaster.ca, accessed on 06/14/2021). ShortBRED-Identify was run with true markers (minimum length eight amino acid residues) filtered against the reference database UniRef90 (https://www.uniprot.org/uniref/, UniProt release 2021_03 on 06/01/21). ShortBRED-Identify identified True Markers as conserved sequences unique to the protein family, whereas Junction Markers shared a slight overlap with other proteins. When specific conserved regions could not be found, ShortBRED-Identify created a Quasi Marker, a peptide sequence with the least overlap to other nonspecific proteins. The marker list used for this study is presented in Table S3.

The marker set included markers for one ARG, *mtrA* (ARO_3000816), which has since been reassigned to an unrelated gene by the CARD curation. Nevertheless, our inspection confirms that our marker sequences do match sequences of the *Mycobacterium* gene *mtrA* (41). The marker sequences labeled *mtrA* were MDTMRQRILVVDDDASLAEMLTIVLRGEGFDTAV, VIGDGTQALTAVRELRPDLVLLDLMLPGMNGIDV, and VCRVLRADSGVPIVMLTAKTDTVDVVLGLESGAD.

To match the target sequences, we ran ShortBRED-Quantify (32) against the trimmed FASTQ files for each sample. A “hit” was recorded whenever (i) USEARCH aligned a read with a marker at 95% identity or greater and (ii) the alignment was at least the entire length of the marker or included 95% of the read length. For each sequenced sample, the relative ARG abundance is the sum of the total hits to all markers for a given ARG.

### Taxa profiles analysis

The DNA sequence FASTQ files were trimmed using Trimmomatic to remove Nextera XT adapters and poor-quality sequences (71). From each filtered sequence, we used Kraken2 with the RefSeq reference database to identify the sequences in our pond samples (34, 72). Kraken2 assigns identity to the sequences in samples of interest by matching *k*-mers in the sample of interest to *k*-mers of the lowest common ancestor (LCA) in a reference database (34). The reference database used was the Kraken2 Standard Refseq Collection containing archaea, bacteria, viral, plasmids, and human sequences, dated June 2023 (https://benlangmead.github.io/aws-indexes/k2, accessed on 09/13/2023). Kraken2 (v2.1.2) first hashed the Standard RefSeq reference database containing archaeal, bacterial, viral, and human genome sequences (accessed June 2023) to reduce active memory usage. Using a sliding window algorithm, Kraken2 then identified memory-minimizing subsequences, so-called *l*-mers, from *k*-mers of length 35 for each trimmed short read in our metagenomes. All distinct *l*-mers for a given read were then converted to a hash sequence and queried against the RefSeq hash table for key matches that correspond to the LCA. To compensate for the possibility of false key identification, the read’s true LCA was assigned to the taxon with the greatest number of *l*-mer key matches. For each of our metagenomes, Kraken2 reported the percent abundance of taxa as a function of all sequenced reads at strain-level resolution.

The proportion of identified reads depends on read quality, reference genome completeness, and the region of genomic DNA encapsulated by the read. Therefore, a fair proportion of reads go unassigned, making Kraken2 output interpretation difficult. We employed Bracken v2.9 to calculate the relative percent abundance of genera using only Kraken2 assigned reads (35). We omitted “human” sequence results because the values appeared inconsistent and increased the variability of overall relative abundance predictions. We used Bracken (Bayesian Re-estimation of Abundance with Kraken2) to compute the relative abundance of identified organisms in our samples (34–36). Since the LCA will often be assigned to higher taxonomy levels, Bracken implemented Bayesian probabilities to determine the likelihood that any given read should be re-classified as some lower taxon.

### Statistical analysis

All statistical analyses were performed using nonparametric statistical techniques in R statistical software (v4.1.2; R Core Team 2021). Spearman correlations were performed with base R (73). Correlations were performed within each pond and across all four ponds. We explored the relationship between tannin concentration and pH using linear regression with Jaeckel’s dispersion function using Rfit v0.27. Heat maps of each correlation table were made with blue representing lowest values and yellow representing highest values. Using vegan v2.7, we employed Nonmetric Multidimensional Scaling, a multi-dimensional ordination technique, to determine which environmental factors were most highly associated with prevalent genera (59–61).

## Data Availability

All metagenome FASTQ files have been deposited at NCBI under SRA accession number PRJNA1107813.

## SUPPLEMENTAL MATERIAL

Supplemental tables and figures spectrum.03034-24-s0001.pdf Tables S1 to S5 and Figure S1.

Table S3 spectrum.03034-24-s0002.pdf ShortBRED markers.

ASM does not own the copyrights to Supplemental Material that may be linked to, or accessed through, an article. The authors have granted ASM a non-exclusive, world-wide license to publish the Supplemental Material files. Please contact the corresponding author directly for reuse.
