# Supplementary material for: Pond water microbiome antibiotic resistance genes vary seasonally with environmental pH and tannins
Source: Microbiol Spectr. 2025 Mar 25;13(5):e03034-24. doi: 10.1128/spectrum.03034-24 (PMC12054064; doi:10.1128/spectrum.03034-24)
Supplement: Supplemental tables and figures — Tables S1 to S5 and Figure S1. [file spectrum.03034-24-s0001.pdf]

Vaccaro et al. 2024. Table S1. Metadata for 2021

Date	Pond	Latitude	Longitude	Field pH	Conductivity (µS/cm)	Water Temperature	DO mg/L	Tannins mg/L	PO4, mg/L	Nitrate, NO3 - mg/L	Ammonia, NH4 mg/L	Water Vegetation	Species	Leaf debris input	Species	Air Temperature (°C)	Weather Conditions	Wind Direction	Wind Speed (mph)	Humidity (%)	Notes
6/9/2021	Burtnett	40°20'58" N	82°19'31" W	6.82	145	23.7	4.05	2	2.51	0	0.02	yes	duckweed, cattails,	yes	oak	22.8	cloudy	northwest	6	80	has been raining for past few days, dogs, sheep
6/9/2021	Burtnett	40°20'58" N	82°19'31" W	6.82	145	23.7	4.05	2.3	2.59	0	0.01	yes	duckweed, cattails,	yes	oak	22.8	cloudy	northwest	6	80	has been raining for past few days, dogs, sheep
6/16/2021	Burtnett	40°20'58" N	82°19'31" W	7.33	107	19.3	7.17	1.5	1.9	0	0.01	yes	duckweed, cattails,	yes	oak	23.3	sunny	southeast	9	36	minnows, sheep, dogs
6/16/2021	Burtnett	40°20'58" N	82°19'31" W	7.33	107	19.3	7.17	1.3	1.9	0.2	0.01	yes	duckweed, cattails,	yes	oak	23.3	sunny	southeast	9	36	minnows, sheep, dogs
6/23/2021	Burtnett	40°20'58" N	82°19'31" W	7.53	97	21.6	9.04	1.4	2.25	0	0.03	yes	duckweed, cattails,	yes	oak	23	sunny	northeast	5	39	minnows, sheep, dogs
6/23/2021	Burtnett	40°20'58" N	82°19'31" W	7.53	97	21.6	9.04	1.3	2.43	0.2	0.05	yes	duckweed, cattails,	yes	oak	23	sunny	northeast	5	39	minnows, sheep, dogs
6/30/2021	Burtnett	40°20'58" N	82°19'31" W	7.88	122	26.4	8.97	1.9	2.89	0	0	yes	duckweed, cattails,	yes	oak	27	partially cloudy	east	5	71	minnows, sheep, dogs, mineral analysis on Friday due to illness
6/30/2021	Burtnett	40°20'58" N	82°19'31" W	7.88	122	26.4	8.97	1.9	3.04	0	0.01	yes	duckweed, cattails,	yes	oak	27	partially cloudy	east	5	71	minnows, sheep, dogs, mineral analysis on Friday due to illness
7/7/2021	Burtnett	40°20'58" N	82°19'31" W	9.4	156	28.7	12.58	1	2.97	0.1	0.04	yes	cattails, low viny vegetation	yes	oak	28	partially cloudy	northeast	3	66	minnows, snails, dogs, evidence of water birds and raccoons
7/7/2021	Burtnett	40°20'58" N	82°19'31" W	9.4	156	28.7	12.58	1.1	3.15	0.1	0	yes	cattails, low viny vegetation	yes	oak	28	partially cloudy	northeast	3	66	minnows, snails, dogs, evidence of water birds and raccoons
7/14/2021	Burtnett	40°20'58" N	82°19'31" W	7.14	112	26.5	8.87	1.8	1.51	0	0.06	yes	cattails, low viny vegetation	yes	oak	26	cloudy	northeast	10	73	minnows, snails, dogs
7/14/2021	Burtnett	40°20'58" N	82°19'31" W	7.14	112	26.5	8.87	1.7	1.49	0	0.03	yes	cattails, low viny vegetation	yes	oak	26	cloudy	northeast	10	73	minnows, snails, dogs
7/21/2021	Burtnett	40°20'58" N	82°19'31" W	6.87	106	22.6	7.18	1.7	2.71	0.1	0.04	yes	cattails, low viny vegetation, white flowers	yes	oak	24	cloudy	south	9	61	minnows, snails, dogs, geese, ducks, water more full, white flowers
7/21/2021	Burtnett	40°20'58" N	82°19'31" W	6.87	106	22.6	7.18	1.8	2.18	0	0.01	yes	cattails, low viny vegetation, white flowers	yes	oak	24	cloudy	south	9	61	minnows, snails, dogs, geese, ducks, water more full, white flowers
7/28/2021	Burtnett	40°20'58" N	82°19'31" W	8.34	118	25	10.65	2.2	2.36	0	0.05	yes	cattails, low viny vegetation, white flowers,algae	yes	oak	30	sunny	southeast	5	51	minnows, snails, dogs, geese, ducks, water up to cattails, green algae
7/28/2021	Burtnett	40°20'58" N	82°19'31" W	8.34	118	25	10.65	2.2	2.36	0	0.05	yes	cattails, low viny vegetation, white flowers,algae	yes	oak	30	sunny	southeast	5	51	minnows, snails, dogs, geese, ducks, water up to cattails, green algae
9/7/2021	Burtnett	40°20'58" N	82°19'31" W	6.08	91	19.8	6.06	2.3	1.45	0	0.05	yes	cattails, low viny vegetation, white flowers,algae	yes	oak	28	sunny	northeast	8	58	minnows, snails, vultures, sheep, dogs
9/7/2021	Burtnett	40°20'58" N	82°19'31" W	6.08	91	19.8	6.06	2.2	1	0	0.05	yes	cattails, low viny vegetation, white flowers,algae	yes	oak	28	sunny	northeast	8	58	minnows, snails, vultures, sheep, dogs
10/5/2021	Burtnett	40°20'58" N	82°19'31" W	6.97	100	20	9.07	3	0.48	0	0.07	yes	cattails, low viny vegetation, white flowers,algae	yes	oak	14	foggy	southwest	0	100	minnows, snails, dogs, sheep, ducks
10/5/2021	Burtnett	40°20'58" N	82°19'31" W	6.97	100	20	9.07	2.3	0.53	0	0.07	yes	cattails, low viny vegetation, white flowers,algae	yes	oak	14	foggy	southwest	0	100	minnows, snails, dogs, sheep, ducks
6/7/2021	Foundation	40°23'10" N	82°29'49" W	8.9	590	24.6	9	0.3	0.06	0.3	0	yes	algae	yes	willow	25	raining/thunder	northeast?	8	70	large amount of geese and goose poop, large fish in water
6/7/2021	Foundation	40°23'10" N	82°29'49" W	8.9	590	24.6	9	0.4	0.15	0	0	yes	algae	yes	willow	25	raining/thunder	northeast?	8	70	large amount of geese and goose poop, large fish in water
6/14/2021	Foundation	40°23'10" N	82°29'49" W	8.21	584	26.5	10.23	0	0.05	0	0	yes	algae	yes	willow	28.3	partially cloudy	southeast	13	49	large amount of geese and goose poop, large fish in water
6/21/2021	Foundation	40°23'10" N	82°29'49" W	8.22	561	26	8.41	0.2	0.03	0.1	0	yes	duckweed, cattails,	yes	willow	26	partially cloudy	southeast	13	65	turtles, evidence of geese
6/21/2021	Foundation	40°23'10" N	82°29'49" W	8.22	561	26	8.41	0	0.04	0	0	yes	duckweed, cattails,	yes	willow	26	partially cloudy	southeast	13	65	turtles, evidence of geese
6/28/2021	Foundation	40°23'10" N	82°29'49" W	8.3	560	28.7	9.6	0.3	0.03	0.2	0	yes	algae	yes	willow	31	sunny	northeast	4	57	turtles, bass, bluegill, geese
6/28/2021	Foundation	40°23'10" N	82°29'49" W	8.3	560	28.7	9.6	0.3	0.09	0.2	0	yes	algae	yes	willow	31	sunny	northeast	4	57	turtles, bass, bluegill, geese
7/5/2021	Foundation	40°23'10" N	82°29'49" W	8.26	544	27.6	10.12	0.4	0.06	0.2	0	yes	duckweed, cattails,	yes	willow	31	sunny	northeast	8	57	turtles, bass, bluegill, geese
7/5/2021	Foundation	40°23'10" N	82°29'49" W	8.26	544	27.6	10.12	0.4	0.09	0.2	0	yes	duckweed, cattails,	yes	willow	31	sunny	northeast	8	57	turtles, bass, bluegill, geese
7/12/2021	Foundation	40°23'10" N	82°29'49" W	8.31	525	26	9.75	0.2	0.02	0	0	yes	algae, submerged tree	yes	willow	28	cloudy	south	8	74	geese - swam through sampling site
7/12/2021	Foundation	40°23'10" N	82°29'49" W	8.31	525	26	9.75	0.4	0.27	0.2	0	yes	algae, submerged tree	yes	willow	28	cloudy	south	8	74	geese - swam through sampling site
7/19/2021	Foundation	40°23'10" N	82°29'49" W	8.18	531	26.7	8.77	0.3	0.06	0.3	0	yes	algae	yes	willow	28	partially cloudy	south	6	51	geese, osprey
7/19/2021	Foundation	40°23'10" N	82°29'49" W	8.18	531	26.7	8.77	0.2	0.09	0.1	0	yes	algae	yes	willow	28	partially cloudy	south	6	51	geese, osprey
7/26/2021	Foundation	40°23'10" N	82°29'49" W	8.25	529	28	9.56	0.2	0.06	0.3	0	yes	algae, submerged tree	yes	willow	30	sunny	west	5	67	geese, osprey
7/26/2021	Foundation	40°23'10" N	82°29'49" W	8.25	529	28	9.56	0.2	0.08	0.2	0	yes	algae, submerged tree	yes	willow	30	sunny	west	5	67	geese, osprey
10/19/2021	Foundation	40°23'10" N	82°29'49" W	7.8	561	17.9	7.92	0.4	0.69	0.1	0.03	yes	milfoil? Algae mats	yes	willow	14	clear, sunny	northwest	5	61	carp, turtles, bass, geese
10/19/2021	Foundation	40°23'10" N	82°29'49" W	7.8	561	17.9	7.92	0.8	0.05	0.3	0.03	yes	milfoil? Algae mats	yes	willow	14	clear, sunny	northwest	5	61	carp, turtles, bass, geese
11/9/2021	Foundation	40°23'10" N	82°29'49" W	7.73	559	11.6	9.6	0.3	0.02	0.3	0.02	yes	milfoil? Algae mats	yes	willow	18	clear, sunny	northeast	8	46	minnows
11/16/2021	Foundation	40°23'10" N	82°29'49" W	7.73	559	11.6	9.6	0.3	0.15	0.2	0	yes	milfoil? Algae mats	yes	willow	18	clear, sunny	northeast	8	46	minnows
11/16/2021	Foundation	40°23'10" N	82°29'49" W	7.9	568	9.5	10.11	0.3	0.04	0.2	0	yes	milfoil? Algae mats	yes	willow	9	cloudy	northwest	3	64	geese

**11/16/2021**	**Foundation**	40°23'10" N	82°29'49" W	7.9	568	9.5	10.11	0.5	0.04	0.2	0	yes	milfoil? Algae mats	yes	willow	9	cloudy	northwest	3	64	geese
**6/7/2021**	**McManis**	40°23'53" N	82°24'24" W	8.65	200	25.5	10.1	0.4	0.03	0	0.03	yes	duckweed, algae	yes	willow, oa	25	sunny	northeast?	8	70	peacock, duck,goat, around pond
**6/7/2021**	**McManis**	40°23'53" N	82°24'24" W	8.65	200	25.5	10.1	0.5	0.6	0.3	0	yes	duckweed, algae	yes	willow, oa	25	sunny	northeast?	8	70	peacock, duck,goat, around pond
**6/14/2021**	**McManis**	40°23'53" N	82°24'24" W	8.38	196	26	10.81	0	0.07	0.3	0	yes	duckweed, algae	yes	willow, oa	28.3	partially cloudy	southeast	13	49	peacock, duck,goat, around pond
**6/14/2021**	**McManis**	40°23'53" N	82°24'24" W	8.38	196	26	10.81	0	0	0.1	0.02	yes	duckweed, algae	yes	willow, oa	28.3	partially cloudy	southeast	13	49	peacock, duck,goat, around pond
**6/21/2021**	**McManis**	40°23'53" N	82°24'24" W	8.85	202	25.8	9.65	0.4	0.07	0.1	0.01	yes	duckweed, algae	yes	willow, oa	26	partially cloudy	southeast	13	65	peacock, duck,goat, cat around pond. Minnows in pond
**6/21/2021**	**McManis**	40°23'53" N	82°24'24" W	8.85	202	25.8	9.65	0.6	0.6	0.2	0	yes	duckweed, algae	yes	willow, oa	26	partially cloudy	southeast	13	65	peacock, duck,goat, cat around pond. Minnows in pond
**6/28/2021**	**McManis**	40°23'53" N	82°24'24" W	9.2	266	28.9	9.9	0.4	0.02	0.3	0.02	yes	duckweed, algae	yes	willow, oa	31	sunny	northeast	4	57	peacock, duck,goat, cat around pond. Minnows in pond
**6/28/2021**	**McManis**	40°23'53" N	82°24'24" W	9.2	266	28.9	9.9	0.6	0.15	0.4	0	yes	duckweed, algae	yes	willow, oa	31	sunny	northeast	4	57	peacock, duck,goat, cat around pond. Minnows in pond
**7/5/2021**	**McManis**	40°23'53" N	82°24'24" W	7.58	211	26.5	8.08	0.7	0.11	0.3	0	yes	duckweed, algae	yes	willow, oa	31	sunny	northeast	8	57	peacock, duck,goat, around pond, more duckweed than before
**7/5/2021**	**McManis**	40°23'53" N	82°24'24" W	7.58	211	26.5	8.08	0.8	0.03	0.3	0	yes	duckweed, algae	yes	willow, oa	31	sunny	northeast	8	57	peacock, duck,goat, around pond, more duckweed than before
**7/12/2021**	**McManis**	40°23'53" N	82°24'24" W	7.09	203	24.2	5.8	0.4	0.12	0.2	0.02	yes	duckweed, algae	yes	willow, oa	28	cloudy	south	8	74	peacock, duck,goat, around pond, duckweed
**7/12/2021**	**McManis**	40°23'53" N	82°24'24" W	7.09	203	24.2	5.8	0.4	0.27	0	0.02	yes	duckweed, algae	yes	willow, oa	28	cloudy	south	8	74	peacock, duck,goat, around pond, duckweed
**7/19/2021**	**McManis**	40°23'53" N	82°24'24" W	7.16	211	24.4	10.05	0.5	0.06	0.1	0	yes	duckweed, algae	yes	willow, oa	28	partially cloudy	south	6	51	peacock, duck,goat, around pond, duckweed
**7/19/2021**	**McManis**	40°23'53" N	82°24'24" W	7.16	211	24.4	10.05	0.8	0.13	0	0	yes	duckweed, algae	yes	willow, oa	28	partially cloudy	south	6	51	peacock, duck,goat, around pond, duckweed
**7/26/2021**	**McManis**	40°23'53" N	82°24'24" W	8.99	221	26.2	11.78	0.2	0.09	0.2	0	yes	duckweed, algae	yes	willow, oa	30	sunny	west	5	67	peacock, duck,goat, around pond, duckweed
**7/26/2021**	**McManis**	40°23'53" N	82°24'24" W	8.99	221	26.2	11.78	0.2	0.01	0.2	0	yes	duckweed, algae	yes	willow, oa	30	sunny	west	5	67	peacock, duck,goat, around pond, duckweed
**6/9/2021**	**Porter**	40° 22' 21.118	82° 24' 56.97"	6.71	96	23.4	6.06	0.5	0.07	0.4	0.09	no	n/a	yes	oak, map	22.8	cloudy	northwest	6	80	has been raining for past few days
**6/9/2021**	**Porter**	40° 22' 21.118	82° 24' 56.97"	6.71	96	23.4	6.06	0.6	0.11	0.4	0.1	no	n/a	yes	oak, map	22.8	cloudy	northwest	6	80	has been raining for past few days
**6/16/2021**	**Porter**	40° 22' 21.118	82° 24' 56.97"	6.86	90	22.8	7.72	0.5	0.15	0.4	0	yes	algae	yes	oak, map	23.3	sunny	southeast	9	36	fish in water
**6/16/2021**	**Porter**	40° 22' 21.118	82° 24' 56.97"	6.86	90	22.8	7.72	0.6	0.09	0.2	0	yes	algae	yes	oak, map	23.3	sunny	southeast	9	36	fish in water
**6/23/2021**	**Porter**	40° 22' 21.118	82° 24' 56.97"	7.16	89	22.5	8.42	0.6	0.16	0.2	0.01	yes	algae	yes	oak, map	23	sunny	northeast	5	39	fish in water
**6/23/2021**	**Porter**	40° 22' 21.118	82° 24' 56.97"	7.16	89	22.5	8.42	0.6	0.11	0.2	0	yes	algae	yes	oak, map	23	sunny	northeast	5	39	fish in water
**6/30/2021**	**Porter**	40° 22' 21.118	82° 24' 56.97"	7.15	90	26.3	7.25	0.7	0.18	0	0	yes	algae	yes	oak, map	27	partially cloudy	east	5	71	fish in water, mineral analysis on Friday due to illness
**6/30/2021**	**Porter**	40° 22' 21.118	82° 24' 56.97"	7.15	90	26.3	7.25	0.7	0.01	0.2	0	yes	algae	yes	oak, map	27	partially cloudy	east	5	71	fish in water, mineral analysis on Friday due to illness
**7/7/2021**	**Porter**	40° 22' 21.118	82° 24' 56.97"	7.18	90	26.8	7.49	0.5	0.1	0.2	0.02	yes	algae	yes	oak, map	28	partially cloudy	northeast	3	66	carp, bass, sunfish, freshwater mussels
**7/7/2021**	**Porter**	40° 22' 21.118	82° 24' 56.97"	7.18	90	26.8	7.49	0.5	0.1	0.2	0.03	yes	algae	yes	oak, map	28	partially cloudy	northeast	3	66	carp, bass, sunfish, freshwater mussels
**7/14/2021**	**Porter**	40° 22' 21.118	82° 24' 56.97"	7.02	93	23.7	8.62	0.5	0.15	0.6	0.06	yes	algae	yes	oak, map	26	cloudy	northeast	10	73	carp, bass, sunfish, freshwater mussels
**7/14/2021**	**Porter**	40° 22' 21.118	82° 24' 56.97"	7.02	93	23.7	8.62	0.4	0.05	0.7	0.03	yes	algae	yes	oak, map	26	cloudy	northeast	10	73	carp, bass, sunfish, freshwater mussels
**7/21/2021**	**Porter**	40° 22' 21.118	82° 24' 56.97"	7.29	103	22.1	11.16	0.4	0.16	1.4	0	yes	algae	yes	oak, map	24	cloudy	south	9	61	carp, bass, sunfish, freshwater mussels
**7/21/2021**	**Porter**	40° 22' 21.118	82° 24' 56.97"	7.29	103	22.1	11.16	0.5	0.05	1.2	0	yes	algae	yes	oak, map	24	cloudy	south	9	61	carp, bass, sunfish, freshwater mussels
**7/28/2021**	**Porter**	40° 22' 21.118	82° 24' 56.97"	7.47	108	25	10.8	0.4	0.08	1.5	0.05	yes	algae	yes	oak, map	30	sunny	southeast	5	51	carp, bass, sunfish, freshwater mussels

Vaccaro et al. 2024. Table S2. Metadata for 2022

Date	Pond	Latitude	Longitude	Field pH	Conductivity (uS/cm)	Water Temperature (°C)	DO mg/L	Tannins mg/L	PO4,-P mg/L	Nitrate, NO3- mg/L	Ammonia NH4-N, mg/L	Water vegetation	Species	Leaf debris input	species	Air Temp (°C)	Weather Conditions	Wind Direction	Wind Speed (mph)	Humidity (%)	Notes	DOC
9/13/2022	Burtnett	40°20'58" N	82°19'31" W	6.31	137	18.4	n/a	2	1.44	0	0	yes	duckweed, cattails, grasses	yes	willow	18.9	partially cloudy	east	16	75		
9/13/2022	Burtnett	40°20'58" N	82°19'31" W	6.31	137	18.4	n/a	2	1.44	0	0	yes	duckweed, cattails, grasses	yes	willow	18.9	partially cloudy	east	16	75		
9/20/2022	Burtnett	40°20'58" N	82°19'31" W	6.7	184	16.2	2.41	1.6	0.73	0.1	0.05	yes	cattails, duckweed	yes	willow	21.1	sunny	east	1	84		9.6
9/20/2022	Burtnett	40°20'58" N	82°19'31" W	6.7	184	16.2	2.41	1.6	0.82	0.1	0.04	yes	cattails, duckweed	yes	willow	21.1	sunny	east	1	84		
9/27/2022	Burtnett	40°20'58" N	82°19'31" W	6.03	122	10.72	2.93	2.4	0.77	0	0.06	yes	Duckweed (light)	yes	willow	10	Partly Cloudy	west	28	74.5	Ducks	6.9
9/27/2022	Burtnett	40°20'58" N	82°19'31" W	6.03	122	10.72	2.93	2.8	0.41	0	0.08	yes	Duckweed (light)	yes	willow	10	Partly Cloudy	west	28	74.5	Ducks	
10/4/2022	Burtnett	40°20'58" N	82°19'31" W	6.05	144	8.11	3.95	1.7	0.81	0	0.03	yes, ducl	duckweed	Yes	Ducks	16.1	Scattered Clouds	NNW	6	60		11.0
10/4/2022	Burtnett	40°20'58" N	82°19'31" W	6.05	144	8.11	3.95	0.9	0.24	0.7	0	yes, ducl	duckweed	Yes	Ducks	16.1	Scattered Clouds	NNW	6	60		
10/11/2022	Burtnett	40°20'58" N	82°19'31" W	5.91	196	6.17	4.72	3.5	1.51	0	0.13	N/A	Geese	N/A	N/A	10	Sunny	SSE	1.864	86	More fine sediments	12.0
10/11/2022	Burtnett	40°20'58" N	82°19'31" W	5.91	196	6.17	4.72	3.6	1.75	0	0.07	N/A	Geese	N/A	N/A	10	Sunny	SSE	1.864	86	More fine sediments	
10/18/2022	Burtnett	40°20'58" N	82°19'31" W	5.89	195	6.61	4.21	1.4	0.39	0.2	0.16	Duckwee	Duckweed less abundant			2.78	Cloudy	W	10.56	88		8.2
10/18/2022	Burtnett	40°20'58" N	82°19'31" W	5.89	195	6.61	4.21	1.8	0.41	1.3	0.19	Duckwee	Duckweed less abundant			2.78	Cloudy	W	10.56	88		
10/25/2022	Burtnett	40°20'58" N	82°19'31" W	6.18	197	9	3.1	1.5	0.68	1.7	0.1	yes				9.44	partly cloudy	SE	10	44%		11.6
10/25/2022	Burtnett	40°20'58" N	82°19'31" W	6.18	197	9	3.1	1.6	1.03	2	0.33	yes				9.44	partly cloudy	SE	10	44%		
11/1/2022	Burtnett	40°20'58" N	82°19'31" W	6.34	226	13.1	2.42	1.9	0.81	2.4	0.8	Loss of D	Large Flock of Geese			13.9	Cloudy	WSW	9.32	95%		8.8
11/1/2022	Burtnett	40°20'58" N	82°19'31" W	6.34	226	13.1	2.42	1.9	0.82	2	0.68	Loss of D	Large Flock of Geese			13.9	Cloudy	WSW	9.32	95%		
11/8/2022	Burtnett	40°20'58" N	82°19'31" W	5.9	270	5.39	4.33	2.2	0.63	0	0.04	getation	low tide, low sediment			6.67	Sunny, Slight Wind	NNE	11	68%		12.9
11/15/2022	Burtnett	40°20'58" N	82°19'31" W	6.07	180	3.1	3.4	3.6	0.53	0	0.17	Pond mo	Ducks			2.78	Cloudy, Light Rain	ESE	7	75%		8.0
11/15/2022	Burtnett	40°20'58" N	82°19'31" W	6.07	180	3.1	3.4	4	0.66	0	0.22	Pond mo	Ducks			2.78	Cloudy, Light Rain	ESE	7	75%		
9/13/2022	Foundation	40°23'10" N	82°29'49" W	8.1	512	22.3	n/a	0	0.01	0	0.03	yes	milfoil(?) some sort of fanned aquatic plant	no		18.9	partially cloudy	east	16	75		
9/13/2022	Foundation	40°23'10" N	82°29'49" W	8.1	512	22.3	n/a	0	0.01	0	0.03	yes	milfoil(?) some sort of fanned aquatic plant	no		18.9	partially cloudy	east	16	75		
9/20/2022	Foundation	40°23'10" N	82°29'49" W	8.34	561	23.1	8.4	0.3	0.05	0.3	0	yes	green algae-like plant	no		21.1	sunny	east	1	84		2.5
9/20/2022	Foundation	40°23'10" N	82°29'49" W	8.34	561	23.1	8.4	0.2	0.13	0.4	0.03	yes	green algae-like plant	no		21.1	sunny	east	1	84		
9/27/2022	Foundation	40°23'10" N	82°29'49" W	8.12	524	18.67	7.97	0.2	0.08	0	0	yes	Algae like plant	no		10	Partly Cloudy	west	28	74.5	osprey, ducks	2.3
9/27/2022	Foundation	40°23'10" N	82°29'49" W	8.12	524	18.67	7.97	0.2	0.04	0	0.01	yes	Algae like plant	no		10	Partly Cloudy	west	28	74.5	osprey, ducks	
10/4/2022	Foundation	40°23'10" N	82°29'49" W	8.08	638	15.7	8.8	0.2	0.02	0.4	0	Leafy Wa	leaves	Yes	N/A	16.1	Scattered Clouds	NNW	6	60		2.4
10/4/2022	Foundation	40°23'10" N	82°29'49" W	8.08	638	15.7	8.8	0.3	0.02	1	0	Leafy Wa	leaves	Yes	N/A	16.1	Scattered Clouds	NNW	6	60		
10/11/2022	Foundation	40°23'10" N	82°29'49" W	8.21	642	14.5	9.62	0.3	0.06	0.3	0.05	N/A	N/A	N/A	N/A	10	Sunny	SSE	1.864	86		2.4
10/11/2022	Foundation	40°23'10" N	82°29'49" W	8.21	642	14.5	9.62	0.3	0	0.4	0.04	N/A	N/A	N/A	N/A	10	Sunny	SSE	1.864	86		
10/18/2022	Foundation	40°23'10" N	82°29'49" W	7.96	148	11.4	N/A	0.4	0.05	0.5	0.14		DO Machine Broken			2.78	Cloudy	W	10.56	88		2.2
10/18/2022	Foundation	40°23'10" N	82°29'49" W	7.96	148	11.4	N/A	0.4	0.06	0.4	0.1		DO Machine Broken			2.78	Cloudy	W	10.56	88		
10/25/2022	Foundation	40°23'10" N	82°29'49" W	7.12	659	12.8	N/A	0	0	0.3	0.03					9.44	partly cloudy	SE	10	44%		2.2
10/25/2022	Foundation	40°23'10" N	82°29'49" W	7.12	659	12.8	N/A	0	0.08	0.3	0					9.44	partly cloudy	SE	10	44%		
11/1/2022	Foundation	40°23'10" N	82°29'49" W	7.83	666	13.4	9.85	0.3	0.17	0.2	0	Clear Water, Low Sediment				13.9	Cloudy	WSW	9.32	95%		2.0
11/1/2022	Foundation	40°23'10" N	82°29'49" W	7.83	666	13.4	9.85	0.3	0.17	0.2	0	Clear Water, Low Sediment				13.9	Cloudy	WSW	9.32	95%		
11/8/2022	Foundation	40°23'10" N	82°29'49" W	7.91	671	11.83	10.3	0.4	0.03	0.3	0			not a lot of leaf litter		6.67	Sunny, Slight Wind	NNE	11	68%		2.1
11/8/2022	Foundation	40°23'10" N	82°29'49" W	7.91	671	11.83	10.3	0.4	0.06	0.4	0			not a lot of leaf litter		6.67	Sunny, Slight Wind	NNE	11	68%		
11/15/2022	Foundation	40°23'10" N	82°29'49" W	7.66	668	8.56	9.88	0.2	0.05	0.1	0.03					2.78	Cloudy, Light Rain	ESE	7	75%		1.8
11/15/2022	Foundation	40°23'10" N	82°29'49" W	7.66	668	8.56	9.88	0.2	0	0.3	0.03					2.78	Cloudy, Light Rain	ESE	7	75%		
9/14/2022	McManis	40°23'53" N	82°24'24" W	6.6	226	20.8	5.7	0.8	0.08	0.2	0.03	yes	duckweed! covering whole pond	yes	willow, oak, mullberry	18.9	cloudy	south	0	84		4.3
9/14/2022	McManis	40°23'53" N	82°24'24" W	6.6	226	20.8	5.7	0.8	0.08	0.2	0.03	yes	duckweed! covering whole pond	yes	willow, oak, mullberry	18.9	cloudy	south	0	84		
9/22/2022	McManis	40°23'53" N	82°24'24" W	6.56	228	21.4	4.31	0.9	0.03	0.1	0.08	yes	lots of duckweed	yes	willow, oak, mullberry	16.1	sunny	est northwe	13	72		4.3
9/22/2022	McManis	40°23'53" N	82°24'24" W	6.56	228	21.4	4.31	0.8	0	0.1	0.05	yes	lots of duckweed	yes	willow, oak, mullberry	16.1	sunny	est northwe	13	72		
9/28/2022	McManis	40°23'53" N	82°24'24" W	6.38	228	16.1	1.66	0.6	0.09	0.4	0.01	yes	lots of duckweed	yes	willow, oak, mullberry	12.2	Cloudy	northwest	17	77	Ducks, Pigs, Peacocks	4.0
9/28/2022	McManis	40°23'53" N	82°24'24" W	6.38	228	16.1	1.66	0.7	0.07	0.4	0.06	yes	lots of duckweed	yes	willow, oak, mullberry	12.2	Cloudy	northwest	17	77	Ducks, Pigs, Peacocks	

**10/5/2022**	**McManis**	40°23'53" N	82°24'24" W	6.95	286	15.4	2.95	0.7	0	0.4	0.06	Lots of d	duckweed	Yes	Ducks, Pigs, Goats, Dog, Cats, F	17.8	Sunny	NW	3	51		4.1
**10/12/2022**	**McManis**	40°23'53" N	82°24'24" W	7.05	289	14.3	3.2	0.9	0.11	0.2	0.13					17.8	Sunny	SSE	6.214	83		3.8
**10/12/2022**	**McManis**	40°23'53" N	82°24'24" W	7.05	289	14.3	3.2	0.8	0.17	0.2	0.13					17.8	Sunny	SSE	6.214	83		
**10/19/2022**	**McManis**	40°23'53" N	82°24'24" W	7.28	295	10.8	5.56	0	0.04	0.4	0	Duckwee	Ducks	Leaf Debris at Bottom	Willow, Pine, Oak	2.78	Rainy, Cold	W	12	94		3.9
**10/19/2022**	**McManis**	40°23'53" N	82°24'24" W	7.28	295	10.8	5.56	0	0.09	0.1	0	Duckwee	Ducks	Leaf Debris at Bottom	Willow, Pine, Oak	2.78	Rainy, Cold	W	12	94		
**10/26/2022**	**McManis**	40°23'53" N	82°24'24" W	7.34	299	14.1	7.32	0.7	0.07	0	0.03	yes	lots of duckweed (more than last week)	Yes	Lots of leaves and ducks!	12.2	rainy	SSW	9	91%		4.1
**10/26/2022**	**McManis**	40°23'53" N	82°24'24" W	7.34	299	14.1	7.32	0.8	0	0	0.05	yes	lots of duckweed (more than last week)	Yes	Lots of leaves and ducks!	12.2	rainy	SSW	9	91%		
**11/2/2022**	**McManis**	40°23'53" N	82°24'24" W	7.28	151	12	7.37	0.7	0.02	0.2	0.03	Duckwee	Ducks, Pigs nearby	Leaf Debris		6.6	Cloudy, Foggy	SSE	1	99%		4.1
**11/2/2022**	**McManis**	40°23'53" N	82°24'24" W	7.28	151	12	7.37	0.8	0.15	0.1	0	Duckwee	Ducks, Pigs nearby	Leaf Debris		6.6	Cloudy, Foggy	SSE	1	99%		
**11/9/2022**	**McManis**	40°23'53" N	82°24'24" W	6.75	305	11.9	6.73	0.7	0.03	0	0.02	less duckweed				6.11	Sunny, Slight Wind	SSE	5	62%		4.0
**11/9/2022**	**McManis**	40°23'53" N	82°24'24" W	6.75	305	11.9	6.73	0.7	0	0.2	0.2	less duckweed				6.11	Sunny, Slight Wind	SSE	5	62%		
**11/16/2022**	**McManis**	40°23'53" N	82°24'24" W	6.62	303	7.6	7.63	0.7	0.05	0.3	0	Light Du	Ducks and Wild Birds			0.56	Cloudy, Light Snow, W	W	11	84%		3.8
**11/16/2022**	**McManis**	40°23'53" N	82°24'24" W	6.62	303	7.6	7.63	0.6	0.04	0	0.03	Light Du	Ducks and Wild Birds			0.56	Cloudy, Light Snow, W	W	11	84%		
**9/14/2022**	**Porter**	40° 22' 21.11	82° 24' 56.97"	6.24	94	18.6	4.59	0.9	0.04	1.1	0.21	no		yes	oak, pine	18.9	cloudy	south	0	84		2.7
**9/14/2022**	**Porter**	40° 22' 21.11	82° 24' 56.97"	6.24	94	18.6	4.59	0.9	0.06	0.8	0.24	no		yes	oak, pine	18.9	cloudy	south	0	84		
**9/22/2022**	**Porter**	40° 22' 21.11	82° 24' 56.97"	6.62	96	20.6	6.62	0.8	0.03	0.8	0.04	no	some side vegetation, but nothing on water	yes	oak, pine	16.1	sunny	est northwe	13	72		2.7
**9/22/2022**	**Porter**	40° 22' 21.11	82° 24' 56.97"	6.62	96	20.6	6.62	0.8	0.03	0.6	0.16	no	some side vegetation, but nothing on water	yes	oak, pine	16.1	sunny	est northwe	13	72		
**9/28/2022**	**Porter**	40° 22' 21.11	82° 24' 56.97"	6.28	81	14.9	4.97	0.5	0.2	1.1	0.13	yes	Clear, no duckweed but pine needles	yes	oak, pine	12.2	Cloudy	northwest	17	77	Oak/Pine Trees	2.3
**9/28/2022**	**Porter**	40° 22' 21.11	82° 24' 56.97"	6.28	81	14.9	4.97	0.5	0.06	0.5	0.15	yes	Clear, no duckweed but pine needles	yes	oak, pine	12.2	Cloudy	northwest	17	77	Oak/Pine Trees	
**10/5/2022**	**Porter**	40° 22' 21.11	82° 24' 56.97"	7.08	112	13.7	6.55	0.7	0.06	0.3	0.24	clear	N/A	Yes	Blue Heron	18.3	Sunny	NW	3	51		2.4
**10/12/2022**	**Porter**	40° 22' 21.11	82° 24' 56.97"	7.08	110	12.2	6.28	0.7	0.44	0.3	0.25					17.8	Sunny	SSE	6.214	83		2.3
**10/12/2022**	**Porter**	40° 22' 21.11	82° 24' 56.97"	7.08	110	12.2	6.28	0.9	0.25	0.4	0.26					17.8	Sunny	SSE	6.214	83		
**10/19/2022**	**Porter**	40° 22' 21.11	82° 24' 56.97"	6.89	109	8.8	6.68	0	0.13	0.4	0			Leaf Debris at Bottom	Maple, Pine, Oak	3.33	Rainy, Cold	W	12	93		2.4
**10/19/2022**	**Porter**	40° 22' 21.11	82° 24' 56.97"	6.89	109	8.8	6.68	0	0.05	0.3	0			Leaf Debris at Bottom	Maple, Pine, Oak	3.33	Rainy, Cold	W	12	93		
**10/26/2022**	**Porter**	40° 22' 21.11	82° 24' 56.97"	7.12	110	12.6	7.64	1.6	0	0.2	0.01	yes	Plants at the bottom	Yes	Lots of leaves - Pines, Oak, Ma	12.2	rainy	SSW	9	91%		3.6
**10/26/2022**	**Porter**	40° 22' 21.11	82° 24' 56.97"	7.12	110	12.6	7.64	1.5	0.06	0.2	0	yes	Plants at the bottom	Yes	Lots of leaves - Pines, Oak, Ma	12.2	rainy	SSW	9	91%		
**11/2/2022**	**Porter**	40° 22' 21.11	82° 24' 56.97"	5.99	107	10.1	6.51	1.1	0.02	0.2	0	Lots of le	Blue Heron	Lots of Leaf Debris		6.6	Cloudy, Foggy	SSE	1	99%		3.0
**11/2/2022**	**Porter**	40° 22' 21.11	82° 24' 56.97"	5.99	107	10.1	6.51	0.9	0.03	0.1	0	Lots of le	Blue Heron	Lots of Leaf Debris		6.6	Cloudy, Foggy	SSE	1	99%		
**11/9/2022**	**Porter**	40° 22' 21.11	82° 24' 56.97"	6.1	109	9.7	4.71	0.9	0.04	0.4	0	more leaves in pond				7.22	Sunny, Slight Wind	SSE	6	58%		2.7
**11/9/2022**	**Porter**	40° 22' 21.11	82° 24' 56.97"	6.1	109	9.7	4.71	0.8	0.13	0.4	0	more leaves in pond				7.22	Sunny, Slight Wind	SSE	6	58%		
**11/16/2022**	**Porter**	40° 22' 21.11	82° 24' 56.97"	6.08	103	5.8	4.56	0.7	0.1	0.1	0.05	Pond = Blue-ish Green in Color		Lots of Leaves		0.56	Cloudy, Light Snow, W	W	11	81%		2.4
**11/16/2022**	**Porter**	40° 22' 21.11	82° 24' 56.97"	6.08	103	5.8	4.56	0.7	0.12	0.1	0.05	Pond = Blue-ish Green in Color		Lots of Leaves		0.56	Cloudy, Light Snow, W	W	11	81%		
**NA**	**Zymo Standard**																					
**NA**	**Zymo Standard**																					
**NA**	**Zymo Standard**																					
**NA**	**Zymo Standard**																					
**NA**	**Zymo Standard**																					
	**Zymo Standard**																					

Vaccaro et al. 2024. Table S4. P-values for Figure 6. Heat map of Spearman correlations between top-ranked ARGs and chemical measures.

**2021**	**acidity**	**tannin**	**PO4**	**NO3**	**NH4**	**cond**	**temp**	**DO**	**2022**	acidity	tannin	PO4	NO3	NH4	cond	temp	DO
**smeB**	0.03	0.06	0.80	0.61	0.22	0.32	0.02	0.08	**smeB**	0.32	0.5	0.9	0.05	0.07	0.61	0.8	0.12
**mtrA**	0.01	0.00	0.03	0.01	0.64	0.20	0.68	0.00	**mtrA**	0.01	0.01	0	0.91	0	0.31	0	0.01
**OXA-156**	0.72	0.01	0.02	0.03	0.22	0.58	0.62	0.14	**OXA-156**	0	0	0.01	0.31	0.43	0.32	0	0
**OXA-409**	0.00	0.00	0.00	0.02	0.00	0.00	0.04	0.00	**OXA-409**	0	0	0.01	0.54	0	0.14	0.52	0
**SHV-100**	0.35	0.16	0.54	0.77	0.55	0.56	0.00	0.50	**SHV-100**	0.38	0.33	0.47	0.11	0.04	0.36	0.47	0.03
**otrC**	0.09	0.54	0.16	0.15	0.09	0.26	0.71	0.32	**otrC**	0.84	0.46	0.77	0.37	0.06	0.68	0.35	0.16
**Erm(O)-lrm**	0.06	0.03	0.05	0.73	0.18	0.62	0.04	0.18	**Erm(O)-lrm**	0.91	0.55	0.57	0.78	0.62	0.14	0.01	0.64
**CTX-M-25**	0.68	0.29	0.78	0.06	0.03	0.77	0.17	0.74	**CTX-M-25**	0.05	0.01	0	0.08	0.42	0.59	0.21	0.05
**vanRO**	0.55	0.54	0.57	0.83	0.58	0.49	0.00	0.89	**vanRO**	0.75	0.86	0.64	0.43	0.31	0.78	0.9	0.82
**floR**	0.35	0.35	0.25	0.75	0.06	0.41	0.03	0.44	**floR**	0	0.01	0	0.07	0.01	0	0.96	0
**tlrB**	0.73	0.02	0.00	0.76	0.43	0.04	0.98	0.86	**tlrB**	0.29	0.24	0.36	0.64	0.16	0.65	0.05	0.48
**FOX-3**	0.00	0.00	0.00	0.06	0.00	0.00	0.04	0.02	**FOX-3**	0	0	0.06	0.37	0.27	0	0.2	0.01
**OXA-372**	0.00	0.03	0.24	0.57	0.95	0.01	0.84	0.01	**OXA-372**	0.12	0.97	0.09	0.21	0.43	0.04	0.89	0.74
**FRI-1**	0.33	0.88	0.44	0.37	0.43	0.03	0.88	0.16	**FRI-1**	0.62	0.9	0.79	0.73	0.47	0.21	0.38	0.65
**adeH**	0.21	0.00	0.00	0.00	0.04	0.39	0.61	0.44	**adeH**	0.96	0.2	0.16	0.05	0.53	0.88	0.74	0.43
**BUT-1**	0.02	0.04	0.13	0.55	0.03	0.00	0.44	0.85	**BUT-1**	0.36	0.82	0.62	0.04	0.34	0.63	0.55	0.03
**OXA-137**	0.93	0.72	0.33	0.10	0.32	0.08	0.38	0.95	**OXA-137**	0.4	0.21	0.01	0.03	0.54	0.14	0.01	0.34
**efrA**	0.90	0.82	0.88	0.25	0.98	0.65	0.58	0.33	**efrA**	0	0	0.12	0.2	0.01	0	0.03	0
**bcrC**	0.84	0.14	0.13	0.05	0.77	0.08	0.89	0.54	**bcrC**	0.06	0.01	0.15	0.17	0.53	0	0.02	0.26
**OXA-46**	0.38	0.00	0.02	0.18	0.13	0.00	0.02	0.53	**OXA-46**	0.01	0.02	0.02	0.24	0.75	0.58	0.01	0.04

Vaccaro et al. 2024. Table S5. Spearman correlations between ARGs and DOC values in 2022.

ARG	Spearman	P-values
smeB	-0.12	0.47
mtrA	0.13	0.43
OXA-156	-0.45	0
OXA-409	0.42	0.01
SHV-100	-0.18	0.27
otrC	-0.28	0.09
Erm(O)-lrm	-0.18	0.28
CTX-M-25	0.35	0.03
vanRO	0.04	0.79
floR	-0.21	0.21
tlrB	-0.33	0.05
FOX-3	-0.30	0.06
OXA-372	-0.09	0.61
FRI-1	0.02	0.91
adeH	0.21	0.21
BUT-1	0.10	0.55
OXA-137	-0.02	0.91
efrA	-0.53	0
bcrC	-0.25	0.14
OXA-46	0.52	0

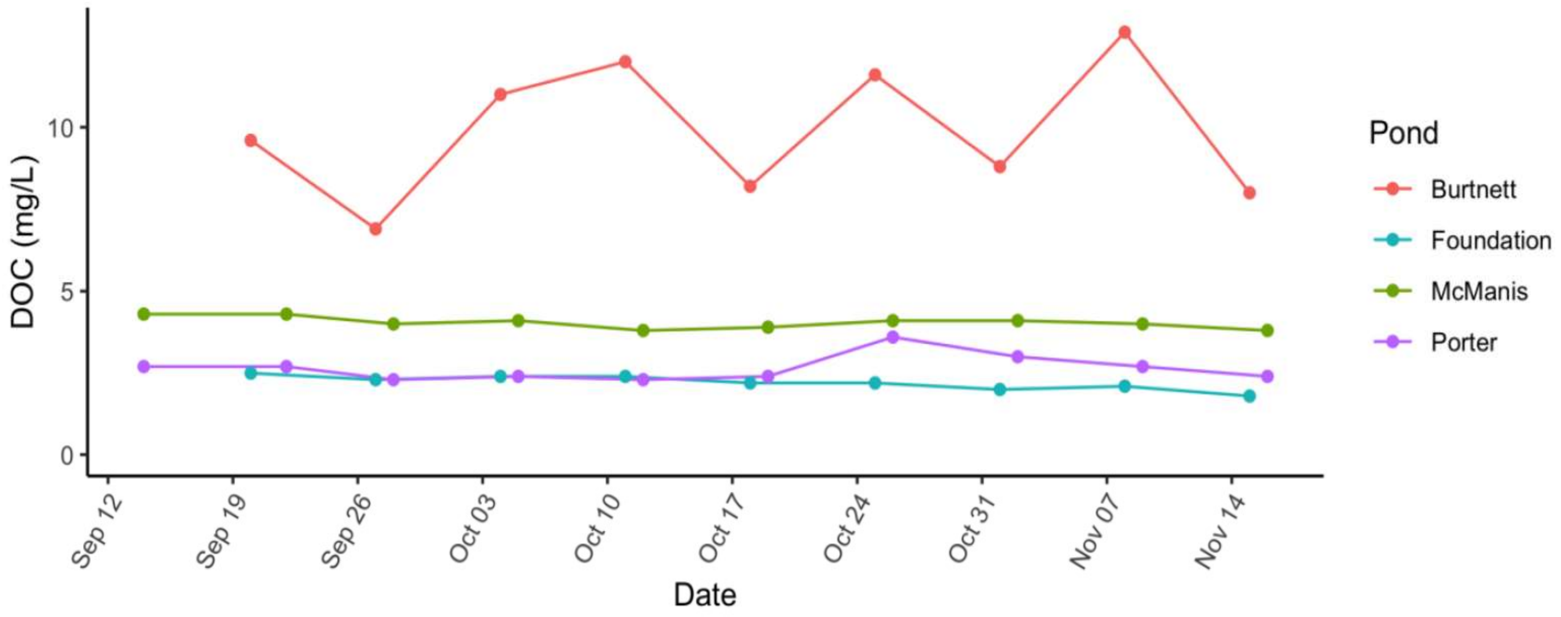

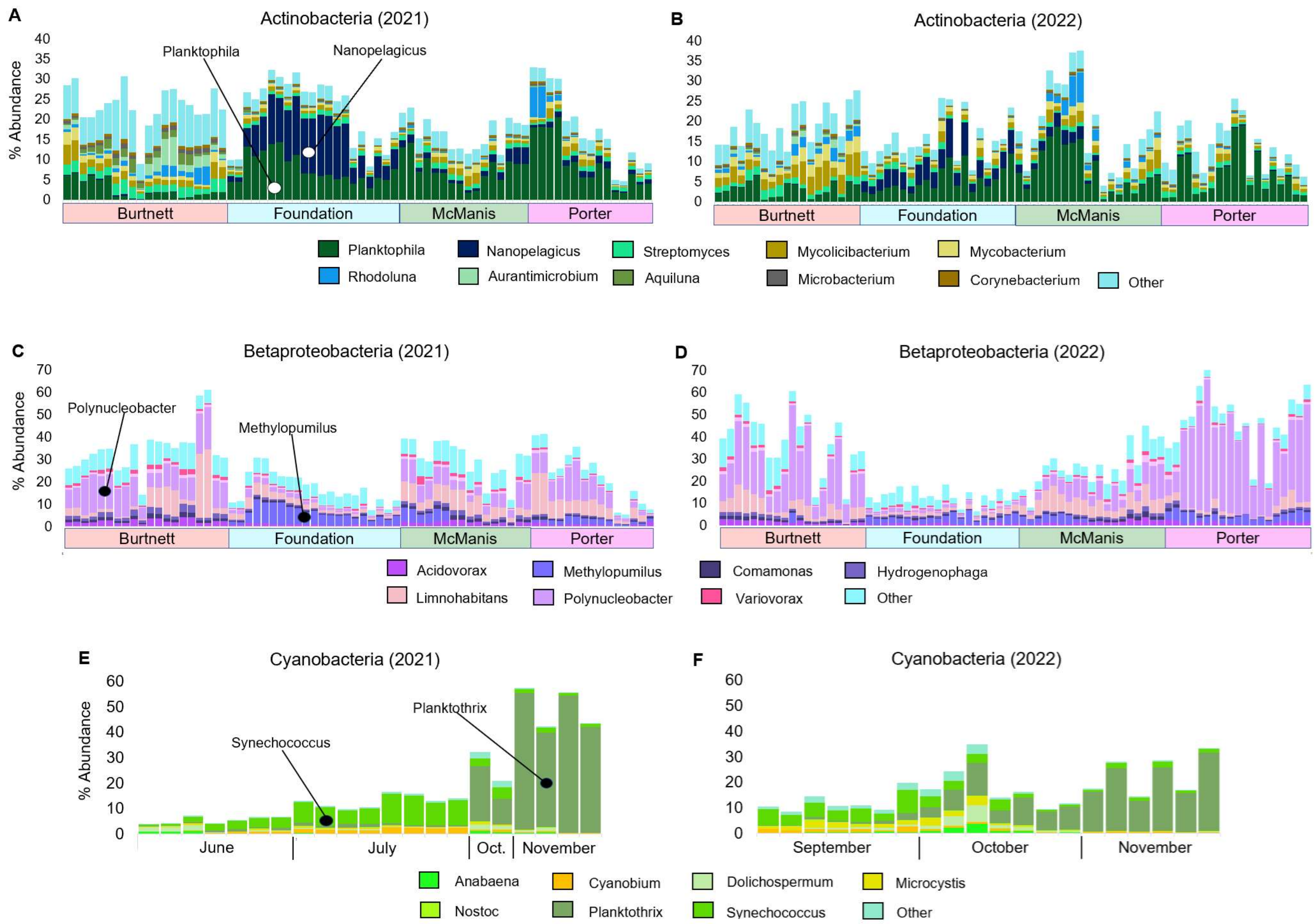

Figure S1. Actinobacteria, Betaproteobacteria, and Cyanobacteria. Genera predicted by Kraken2/Bracken. Genera predicted for: **A**, Actinobacteria in 2021; **B**, Actinobacteria in 2022; **C**, Betaproteobacteria in 2021; **D**, Betaproteobacteria in 2022**; E**, Cyanobacteria in Foundation Pond, 2021; **F**, Cyanobacteria in Foundation Pond, 2022.
